# Laboratory evolution reveals a two-dimensional rate-yield tradeoff in microbial metabolism

**DOI:** 10.1371/journal.pcbi.1007066

**Published:** 2019-06-03

**Authors:** Chuankai Cheng, Edward J. O’Brien, Douglas McCloskey, Jose Utrilla, Connor Olson, Ryan A. LaCroix, Troy E. Sandberg, Adam M. Feist, Bernhard O. Palsson, Zachary A. King

**Affiliations:** 1 Department of Bioengineering, University of California San Diego, La Jolla, California, United States of America; 2 Novo Nordisk Foundation Center for Biosustainability, Technical University of Denmark, Lyngby, Denmark; 3 Department of Pediatrics, Univerity of California San Diego, La Jolla, California, United States of America; Ecole Polytechnique Fédérale de Lausanne, SWITZERLAND

## Abstract

Growth rate and yield are fundamental features of microbial growth. However, we lack a mechanistic and quantitative understanding of the rate-yield relationship. Studies pairing computational predictions with experiments have shown the importance of maintenance energy and proteome allocation in explaining rate-yield tradeoffs and overflow metabolism. Recently, adaptive evolution experiments of *Escherichia coli* reveal a phenotypic diversity beyond what has been explained using simple models of growth rate versus yield. Here, we identify a two-dimensional rate-yield tradeoff in adapted *E. coli* strains where the dimensions are (A) a tradeoff between growth rate and yield and (B) a tradeoff between substrate (glucose) uptake rate and growth yield. We employ a multi-scale modeling approach, combining a previously reported coarse-grained small-scale proteome allocation model with a fine-grained genome-scale model of metabolism and gene expression (ME-model), to develop a quantitative description of the full rate-yield relationship for *E. coli* K-12 MG1655. The multi-scale analysis resolves the complexity of ME-model which hindered its practical use in proteome complexity analysis, and provides a mechanistic explanation of the two-dimensional tradeoff. Further, the analysis identifies modifications to the P/O ratio and the flux allocation between glycolysis and pentose phosphate pathway (PPP) as potential mechanisms that enable the tradeoff between glucose uptake rate and growth yield. Thus, the rate-yield tradeoffs that govern microbial adaptation to new environments are more complex than previously reported, and they can be understood in mechanistic detail using a multi-scale modeling approach.

## Author summary

This study reconciles multiple existing microbial rate-yield tradeoff theories with experimental data. There is great interest in developing quantitative descriptions of the relationship between growth rate and growth yield [1]. However, some reported experiments [2–4] in the literature do not agree with existing theories [5–7]. Specifically, overflow metabolism in *E. coli* can either be coupled [5, 8] or decoupled [2–4] from growth rate. We found that adaptive laboratory evolution (ALE) experiments of *E. coli* reveal a two-dimensional rate-yield tradeoff in adapted strains where the dimensions are (i) a tradeoff between growth rate and growth yield, previously reported by [5], and (ii) a tradeoff between substrate uptake rate and growth yield. The appearance of this two-dimensional tradeoff during adaptation suggests that microorganisms adapting to new environments are subject to a more complex set of rate-yield tradeoffs than previously reported [5, 6]. In this study, the two-dimensional rate-yield tradeoff is quantitatively explained through our multi-scale modeling approach, combining a previously reported small-scale proteome allocation model [5] with a genome-scale model of metabolism and gene-expression (ME-model) [9]. The modeling approach is also instrumental to future studies.

## Introduction

Growth rate and yield are basic features of microbial life that are widely implicated in cell fitness, adaptation, and evolution [1]. The specific growth rate, *μ*, represents the number of doublings of bacterial density per unit time [10]. The yield, *Y*, is the ratio between *μ* and the rate of substrate consumption [10, 11]. The mathematical relation between *μ* and *Y* can be written as:

$$\begin{array}{c} Y=\frac{\mu }{M_{substrate}\cdot q_{substrate}} \end{array}$$ (1)

where *M*_*substrate*_ is the molecular weight of the substrate and *q*_*substrate*_ is the substrate uptake rate.

**Fig 1:**
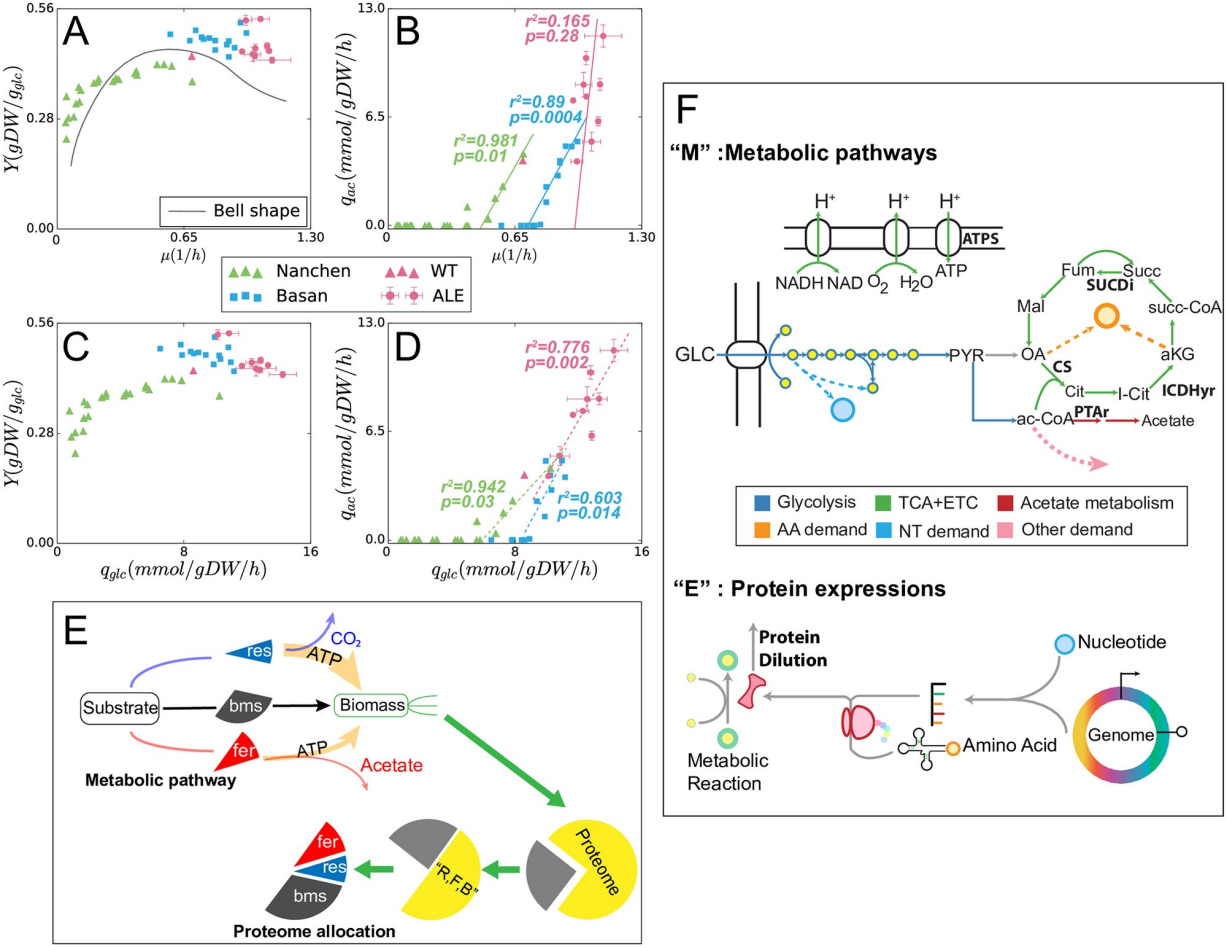
*E. coli* growth phenotypes in minimal media and multi-scale modeling approaches. Data on the plots are recorded in S7–S10 Tables (Supporting Information). (A–D) Phenotypic data for *E. coli* strains including *Y*, *μ*, *q*_*ac*_, and *q*_*glc*_ data. The *Y* is calculated by $\frac{\mu }{M_{glc}\cdot q_{glc}}$, where the molecular weight of glucose *M*_*glc*_ = 180.156*g*/*mol*. Two datasets are presented from literature, for chemostat growth [8] (green triangles) and substrate titration [5] (blue squares). These are compared to strains adapted for maximum growth rate through ALE (this study; red circles; error bars for standard deviation across duplicates). The bell-shaped *μ*–*Y* relationship proposed by [1] is included for reference. (E) Diagram of the SSME-model derived from [5]. The model consists of three pathways: respiration (R, res) and fermentation (F, fer) generate different amounts of energy, feeding the biomass (B, bms) pathway to synthesize biomass.(F) Diagram of the genome-scale ME-model that includes a genome-scale reconstruction of metabolic pathways (“M”) and protein expression machinery (“E”) [9, 16]. Only central carbon metabolism is sketched, other biosynthesis pathways (AA amino acids, NT nucleotides and others) are simplified as dashed arrows in the plot, but remain fine-grained in the genome-scale ME-model that we analyze in this study. For the “E” pathway, it starts with the amino acids and nucleotides that are synthesized through “M” pathway, and the end product of “E” pathway is the dilution of the protein (enzyme). The dilution rate of the enzyme determines the corresponding metabolic reaction rate with the factor *k*_*eff*_. More details about *k*_*eff*_ constraints can be found in “3 Proteome constraints in the ME-model” in S1 Appendix.

In the context of modeling the phenotypic relation between substrate uptake, metabolism, and biomass growth, there is a great interest in developing quantitative descriptions of the relationship between *μ* and *Y*. The wide-ranging measurements of *μ* and *Y* (Fig 1A) across microbial communities and environments raised interest into the exact nature of the *μ*–*Y* relationship [1]. At low *μ*, positive correlations between *μ* and *Y* have been observed [8], and these can be explained by non-growth-associated cell maintenance requirements that make slow growth inefficient [11]. At high *μ*, negative correlations between *μ* and *Y* are observed [5], and for *E. coli*, this can be explained by a tradeoff between metabolic efficiency and enzymatic efficiency that lead to decreasing *Y* at high *μ* [12, 13]. In particular, *E. coli* exhibits a tradeoff between respiration, which has higher energy yield per carbon substrate (more metabolically-efficient), and acetate fermentation, which requires less enzyme per carbon substrate (more proteome-efficient). Therefore, acetate excretion increases linearly with *μ* above a threshold growth rate (green and blue lines in Fig 1B) [5]. [1] summarized these observations where positive *μ*–*Y* correlation at low *μ* and negative *μ*–*Y* correlation at high *μ* are different parts of a bell-shaped *μ*–*Y* curve (Fig 1A). However, recent experiments suggest that adaptation to new environments can modify the bell-shaped *μ*–*Y* tradeoff [3, 14, 15].

Microorganisms rapidly adapting to environmental niches [17, 18], and adaptation mechanisms can be studied directly through adaptive laboratory evolution (ALE) [19]. When strains are adapted through ALE for growth in a liquid minimal medium, they achieve higher *μ* compared to the wild-type (Fig 1A), ALE-adapted strains have been shown to rapidly acquire regulatory mutations that modify proteome allocation, but they do not acquire new metabolic capabilities within the time frame of reported short-term (4 to 8 weeks) adaptation experiments [3, 15, 20]. By analyzing ALE-adapted strains, we can reveal the strategies that allow cells to optimize their proteome allocation for growth in an environmental niche, subject to the constraints of their metabolic capabilities (i.e. their repertoire of pathways) and constraints on the kinetic efficiencies of their enzymes [3, 20, 21].

Contrary to the negative *μ*–*Y* relationship at high *μ* observed in wildtype strains, the endpoint strains of ALE experiments of *E. coli* selected for high *μ* in a minimal medium do not have *μ*–*Y* data points aligning on the bell-shape curve in Fig 1A, but reveal an uncorrelated relationship between *μ* and *Y* [3, 15]. In these experiments, strains exhibited little variation in *μ* but high variation in *Y* and acetate excretion rate *q*_*ac*_. Previous studies similarly reported that overflow metabolism can be nearly eliminated through genetic engineering without any effect on growth rate in *E. coli* [2, 4]. Thus, the negative *μ*–*Y* correlation at high growth rates does not appear to be a fundamental constraint on fast-growing cells. A mechanistic model of the full *μ*–*Y* relationship must be able to reconcile the bell-shaped curve observed for individual strains with the uncorrelated *μ*–*Y* phenotypes seen in ALE-adapted strains (Fig 1A).

A number of theoretical and computational models have been developed to describe rate-yield tradeoffs. For the positive *μ*–*Y* correlation, maintenance requirements can be quantitatively described using algebraic growth laws [8, 11]. The maintenance requirement has been modeled as non-growth associated maintenance (NGAM) in the genome-scale models (GEMs) of metabolism, which can be simulated as an optimization problem, predicting *μ* and *Y* when substrate uptake rates (e.g. *q*_*glc*_) are known [22]. For the negative *μ*–*Y* correlation, quantitative models of overflow metabolism have been developed [5–7]. In particular, quantitative measurements of *E. coli* growth in well-controlled environments revealed a linear-threshold response of acetate excretion (*q*_*ac*_) with increasing *μ* [5]. To represent the full range of the *μ*–*Y* relationship, a constraint allocation flux balance analysis model (CAFBA) was reported that combines a GEM with proteome allocation constraints [6]. A similar solution can be formulated from a bottom-up reconstruction of metabolism and macromolecular expression (ME-model, [9]) that incorporates the protein synthesis pathways into a GEM and applies coupling constraints related to enzyme kinetics parameters on each individual reaction. However, none of these models have been used to explain experiments where *μ* and *Y* are decoupled through laboratory evolution or genetic engineering.

In this study, we show that the wide range of *μ*–*Y* observations in *E. coli* can be explained by a two-dimensional rate-yield tradeoff, where the first dimension is the characteristic *μ*–*Y* tradeoff associated with acetate overflow metabolism and the second dimension is a tradeoff between glucose uptake rate (*q*_*glc*_) and *Y* that appears during ALE adaptation. We employ a multi-scale modeling approach to provide a mechanistic description of the two-dimensional rate-yield tradeoff. By deriving the relationship between the ME-model and the previously reported small-scale proteome allocation model [5], we are able to develop a workflow for modifying ME-model parameters to fit experimental data, and we achieve quantitative predictions for simulations of *μ*–*Y* (the first dimension of the rate-yield tradeoff). This multi-scale modeling approach predicts a two-dimensional rate-yield tradeoff, and it suggests that the second dimension of the tradeoff can be explained by changes in P/O ratio and the flux balance between glycolysis and pentose phosphate pathway. This multi-scale modeling approach predicts the systemic response of the cell to growth selection, representing the relationships between P/O ratio, glycolytic-PPP flux balance, and the two dimensions of the rate-yield tradeoff.

## Results

### Adaptive laboratory evolution reveals a two-dimensional rate-yield tradeoff

To explore the metabolic constraints on *E. coli* growth, adaptive laboratory evolution (ALE) was used to adapt *E. coli* K-12 MG1655 to maximize growth at 37°C in a liquid culture with a minimal medium containing glucose [3]. Eight independent experiments were performed on an automated ALE platform to achieve 8.3 × 10^12^ to 18.3 × 10^12^ cumulative cell divisions [23]. Phenotype characterization was performed on eight ALE endpoint strains, including quantitative measurements of *μ*, *q*_*glc*_, *q*_*ac*_, and other common metabolic byproducts of *E. coli* (Materials and methods).

A diversity of metabolic phenotypes was observed in the ALE endpoint strains. Through ALE, *μ* increased from 0.7 h^-1^ for wild-type (red triangles in Fig 1A–1D) to 0.95–1.10 h^-1^ (red circles with error bars in Fig 1A–1D). Based on previous reports, we expected a linear relationship between *μ* and *q*_*ac*_. However, ALE endpoint strains achieved a wide ranging *q*_*ac*_ from 3.9–11.4 mmol gDW^-1^ h^-1^ (where wild-type *q*_*ac*_ was 3.9 mmol gDW^-1^ h^-1^. While we did not observe a correlation between *μ* and *q*_*ac*_ in these strains (Fig 1B), there was a clear correlation between *q*_*glc*_ and *q*_*ac*_ (Fig 1D).

Two of the ALE endpoint strains with similar *μ* (3% difference) but distinct *Y* (30% difference) have been processed for ^13^C metabolic flux measurements (S11 Table). The measured metabolic fluxes using ^13^C metabolic flux analysis (^13^C MFA, see “Materials and methods”) shows the positive correlation between TCA fluxes, *q*_*TCA*_, and *Y* for the ALE strains. For the ALE strain with larger *Y*, *q*_*glc*_ and *q*_*ac*_ are lower and *q*_*TCA*_ is higher. Therefore, for a fixed *μ*, *Y* increases as *q*_*TCA*_ increases and *q*_*ac*_ decreases, indicating a pathway switch between the TCA cycle and acetate overflow depending on *q*_*glc*_.

Therefore, combining with the referenced study [5], for a wild-type strain, there is a *μ*–*Y* tradeoff. And for the isogenic ALE strains, a *q*_*glc*_–*Y* tradeoff appears. For both tradeoffs, *Y* varies with the pathway switch between TCA cycle and acetate overflow. In this paper, we call the *μ*–*Y* and *q*_*glc*_–*Y* tradeoffs a two-dimension rate-yield tradeoff, since they are tradeoffs between different “rates” (growth rate *μ* and glucose uptake rate *q*_*glc*_) and the same yield (glucose yield *Y*), and they share the same phenotypic behavior of TCA–aceate overflow pathway switch.

Correlations between *q*_*glc*_, *Y*, and *q*_*ac*_ have been observed previously for *E. coli* strains [2–4], and moreover, a bacterial engineering approach has been reported to vary *q*_*ac*_ by manipulating the substrate uptake system [24]. In one of these studies, [4] showed that switching electron transport chain (ETC) enzyme selection (and thereby modifying the P/O ratio) can cause a *q*_*glc*_–*Y* tradeoff at a low *μ* of 0.15 h^-1^. ALE gained *q*_*ac*_ and *Y* decoupled from *μ*, which seemingly differs from the reported correlation between *μ*–*Y* and *μ*–*q*_*ac*_ [5, 8]. The ME-model used in this study simulates the relationships between these *q*_*glc*_–*q*_*ac*_ and *q*_*glc*_–*Y* tradeoffs, connecting to the mechanisms of *μ*–*Y* tradeoffs (the bell-shaped curve in Fig 1A) by established models [5, 6].

To enable our analysis, it is important to note that ALE endpoint strains rapidly acquire regulatory mutations, but they do not acquire new metabolic capabilities within the time frame of these experiments [3, 15, 20]. The linear correlation between *q*_*ac*_ and *μ* reported previously was identified for an isogenic strain [5, 8]. In contrast, our observations of a decoupling between *q*_*ac*_ and *μ* appear when comparing adapted strains. However, because these adapted strains have only regulatory mutations, their phenotypes represent the limits of what *E. coli* cells can achieve while bounded by metabolic and proteomic constraints (but not by regulation). This type of adaptation and the associated phenotypic tradeoffs are useful for understanding cellular adaptation to ecological niches where regulatory adaptation can occur rapidly [18].

### ME-model data fitting with a multi-scale modeling approach

**Fig 2:**
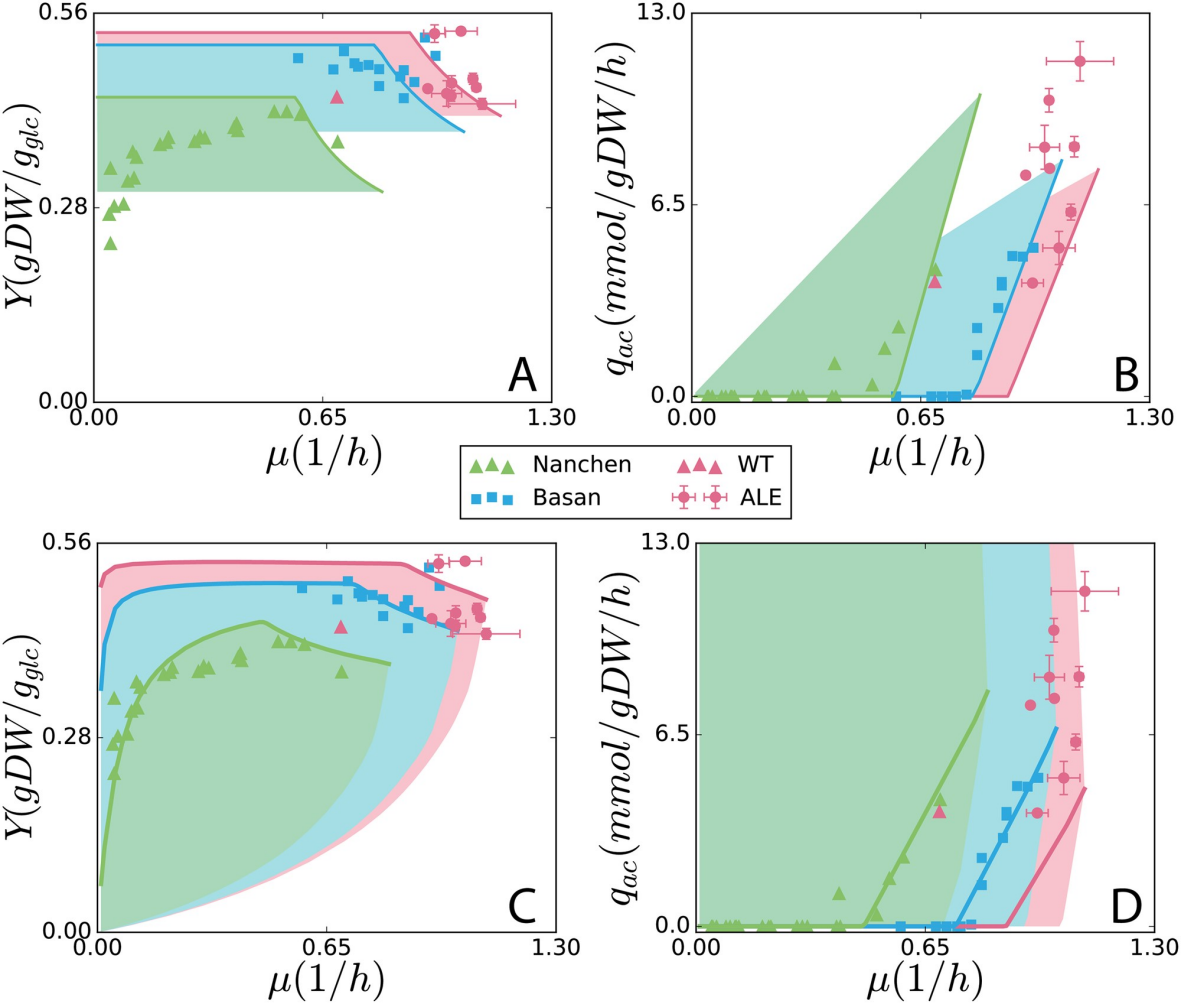
SSME-model and ME-model simulations. Growth phenotypes of *E. coli* from simulations: (A, B) using the SSME-model and (C, D) ME-model. Simulations were fit to experimental data for each of the three datasets, K-12 MG1655 chemostat [8] (green triangles), NCM3722 substrate titration [5] (blue squares), and strains adapted from wild-type K-12 MG1655 (red triangle, [3] for maximum growth rate through ALE (this study, red circles, error bars for standard deviation across duplicates). The *Y*-maximized solutions are displayed as solid lines in all plots. Solution spaces are simulated by taking the feasible range between maximum (*Y*_*max*_–*Y*_*min*_ in A and C, *q*_*ac*,*max*_–*q*_*ac*,*min*_ in B and D)For both models, fitting was performed by manipulating three global parameters: unmodeled protein fraction (UPF), growth-associated maintenance (GAM), and non-growth associated maintenance (NGAM). Details of the fitting approach are provided in Materials and Methods.

To explain these experimental observations, we sought a modeling approach that could quantitatively predict the *μ*–*Y* and *μ*–*q*_*ac*_ relationships. Our modeling approach starts with fitting the linear-threshold (blue line in Fig 1B) *mu*–*q*_*ac*_ relation [5] using the framework of ME-model [9, 16]. We first considered a previously reported coarse-grained model of proteome allocation [5] that describes *E. coli* overflow metabolism (Fig 1E). [5] solves *Y* and *q*_*ac*_ as functions of *μ*, and assuming that the cells pick the maximum *Y* under each particular *μ*. This indicates that high-*Y* growth strategies have a fitness benefit in spatially structured environments, for instance, the wild-type cultures collected from colonies, that has been demonstrated through a *Y*-selection system [14], and more efficient strategies also leave more resources for cells that are hedging against future stresses [20]. The evolutionary history of *E. coli* includes growth in structured environments and a wide range of stresses that could have placed a selection pressure on increasing *Y*. Therefore, we focused on fitting the observed wild-type chemostat [8] and uptake titration [5] data for the *Y*-maximized growth solution (green and blue data points in Fig 2).

The coarse-grained proteome allocation model [5] was intended to make predictions at high *μ* and thus only captures the negative *μ*–*Y* relation (Fig 2A). The parameters in the coarse-grained model have a strong experimental basis in fine-grained protein abundances measurements in high growths, and the model simulates accurate predictions of *μ*–*q*_*ac*_ [5].

We also considered the genome-scale ME-model *i*JL678-ME [16]. With the default parameter settings in the ME-model, simulations had a poor quantitative prediction [9] of *μ*–*q*_*ac*_ to the uptake titration data (S3F Fig). As [5] has shown experimentally that the overflow metabolism is fundamentally caused by the tradeoff between metabolic efficiency (reaction stoichiometry) and protein efficiency (enzyme turnover rate). Since the reaction stoichiometry in the ME-model has been mass-balanced and well established, we suspect that this poor fit from the ME-model can be explained by inaccurate genome-wide enzyme turnover rates (*k*_*eff*_s) that ME-model researchers have been seeking to improve [16, 25, 26]. We sought to modify the *k*_*eff*_s to fit the *μ*–*q*_*ac*_ data. However, since each of the 5266 reactions in the genome-scale ME-model has a *k*_*eff*_ parameter, it is difficult to directly fit the parameters to measured data.

Therefore, we pursued a multi-scale modeling approach where the coarse-grained model was used to analyze the effects of proteome-efficiency at the level of coarse-grained pathways instead of each individual reaction, which helps to tune the fine-grained parameters in the ME-model. To connect the coarse-grained and fine-grained models, we first found that the proteome efficiency (*ε*) parameters in the coarse-grained model share a conceptual basis with the enzyme efficiency parameter *k*_*eff*_s in ME-models (“3 Proteome constraints in the ME-model” in S1 Appendix). Thus, we were able to reformulate the coarse-grained model within the framework as the ME-model (S1 Fig). The resulting small-scale ME-model (SSME-model) has parameters directly analogous to those in the genome-scale ME-model (See “5 SSME-model parameters derivation” and “6 Matlab and COBRAme implementation” in S1 Appendix). The resulting SSME-model generates identical *μ*–*Y* and *μ*–*q*_*ac*_ predictions to the proteome allocation model.

The SSME-model is a good tool for *k*_*eff*_ parameter sensitivity analysis [27], which provides insights on how to modify the *k*_*eff*_ of the ME-model to achieve quantitative fit. As a result, we gained predictions for *μ*–*Y* and *μ*–*q*_*ac*_ from both the SSME- and ME-models (blue curves in Fig 2). Details of the ME-model modifications are in the S1 Appendix (“8 Experimental data fitting”). In summary, with the multi-scale modeling approach, we identified the reactions whose enzymes turnover rates are too high to match the observed phenotypes. Those reactions are involved in different pathways, including the TCA cycle, Entner-Doudoroff pathway, glyoxylate shunt, nucleotide salvage, and fatty acids metabolism (S3 Table). Three global parameters, unmodeled protein fraction (UPF), growth-associated maintenance (GAM), and non-growth-associated maintenance(NGAM) (S2 Table) were then used to predict the phenotype from different strains (green, blue and red curves in Fig 2).

The reason for only modifying global parameters to simulate the ALE adaptation is that the mutations in the ALE strains do not directly related to the enzyme turnover rate (*k*_*eff*_ value) of a particular metabolic reaction. According to previous ALE studies [3], most mutations occur in genes associated with regulations or translations. Even in the cases where mutations might directly change a *k*_*eff*_, this is hard to model. Therefore, rather than exploring the mechanistic effects of ALE mutations, we focused on the phenotypic changes in the endpoint strains. Some recent studies have shown how individual mutations can have wide-reaching effects on gene expression, metabolic pathway activity, and cell phenotype [3, 20].

The most obvious difference between the SSME-model derived from [5] and ME-model for these phenotypic predictions is the expanded solution space of the ME-model (Fig 2). However, much of the ME-model solution space corresponds to very low yield metabolic solutions. If *Y* is maximized during simulations of the SSME-model and ME-model (achieved by minimizing *q*_*glc*_ at a given *μ*), the resulting predictions are more similar between the models and lie closer to experimental data (solid blue curves in Fig 2). For the growth-rate-dependent *Y*-maximized solutions (solid curves in all 4 panels), though the *q*_*ac*_ lines looks similar, the *Y* lines looks very different in the low growth regime. Where the SSME-model predicts a constant high *Y*, the ME-model predicts an initially low *Y* that increases rapidly with *μ*. This is because the additional non-growth maintenance energy (NGAM) added in the ME-model. We can also see that in Fig 2C, among the three different solution curves from the ME-model, the curve with lower NGAM has a higher *Y* at low *μ*. The NGAM parameters for the different curves are shown in S1 Table in Supporting information. In fact, by adding complexity to the SSME-model or simplifying the ME-model, many intermediate models can be built.

### The ME-model predicts phenotypic diversity in ALE strains

As a result of data fitting, we achieved a quantitative fit of chemostat [8] and batch [5] uptake titration data with the *Y*-maximized ME-model solutions (blue and green curves in Fig 2C and 2D). The ALE-adapted strains (red circles in Fig 2) do not align well with the *Y*-maximizing solutions (red curves in Fig 2), but they are encompassed by the ME-model solution space. Further analysis of these ALE data points and the corresponding ME-model solutions were used to understand the phenotypic diversity of these adapted strains.

Feasible solutions other than the *Y*-maximized solution are achieved through the activation of alternative metabolic pathways which are sub-optimal. The SSME-model does not capture the ALE data points with high *q*_*ac*_ (red region in Fig 2B), while the genome-scale ME-model does (red region in Fig 2D). Moreover, the ME-model predicts feasible growth at lower *Y* in the *μ*–*Y* solution space than the SSME-model. We sought to determine which pathways are responsible for the lower *Y* and higher *q*_*ac*_ in ME-model that was not captured by the SSME-model.

Removing reactions from the ME-model can decrease the size of the solution space (S5, S6 and S7 Figs, “8 Solution space variation” in S1 Appendix), making the solution space more similar to the SSME-model solution space. We employed a workflow to identify 24 reactions (S6 Table) that are not activated in the *Y*-maximized solutions but are used to enable higher *q*_*ac*_ at lower *Y*. We observed that these 24 reactions are part of metabolically inefficient pathways that are alternatives to the *Y*-optimal pathways. By extension, metabolically inefficient pathways can be added to the SSME-model to increase the size of the solution space (S7 Fig), making it more similar to the ME-model solution space. Thus, the modified SSME-model can achieve low *Y* (S7A Fig) at high *q*_*ac*_ (S7C Fig). Therefore, the difference in predictions of ME-model from the SSME-model is a result of the greater range of metabolic capabilities of the genome-scale model.

### The two-dimensional rate-yield tradeoff

We can now put forward a theory to connect the correlations in *μ*–*Y* (Fig 1A) (and the associated acetate curve in *q*_*ac*_-–*Y*, Fig 1B) with the negative correlation in *q*_*glc*_-–*Y* (Fig 1C) and positive *q*_*glc*_—*q*_*ac*_ correlations (Fig 1D).

**Fig 3:**
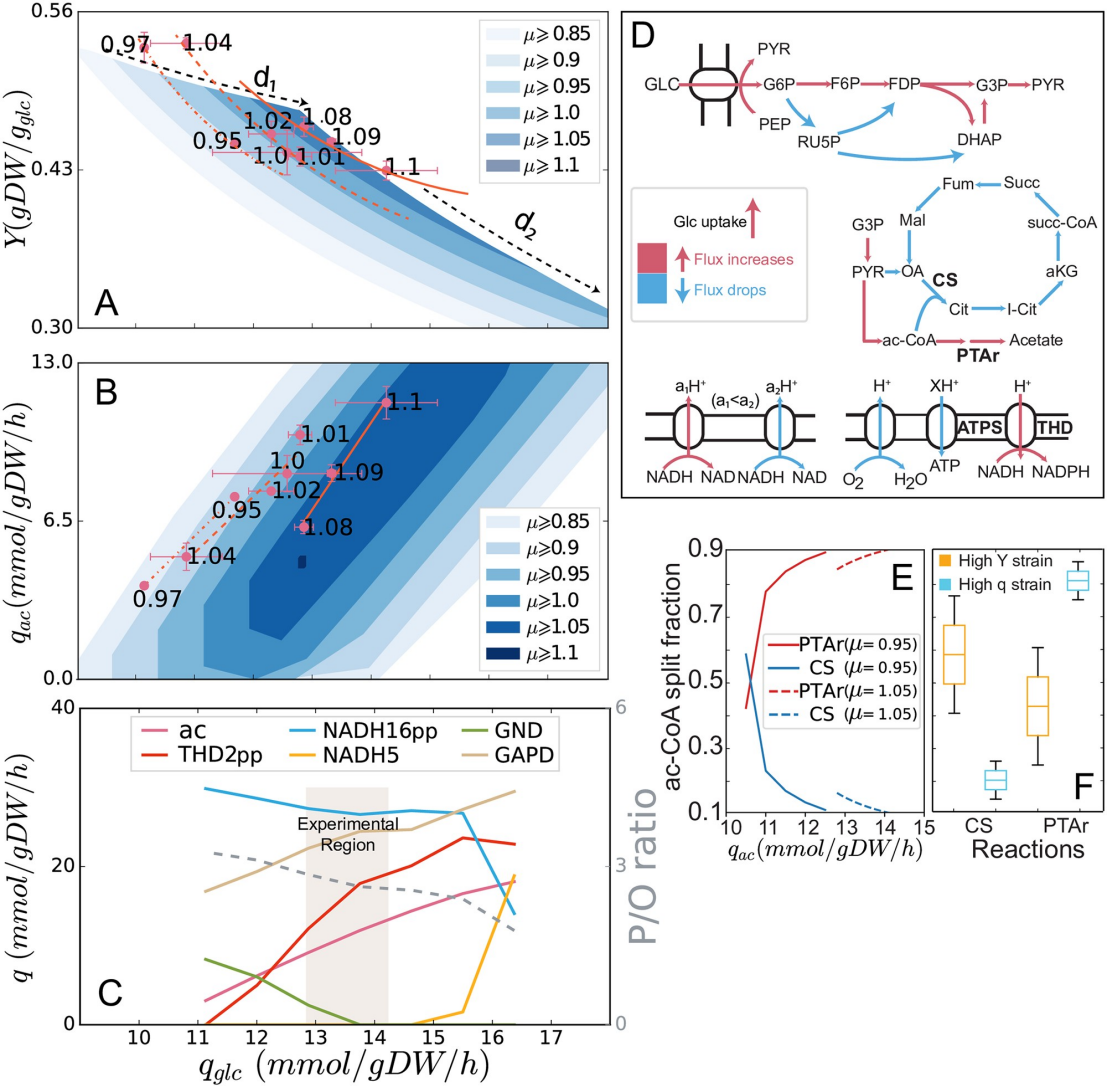
Analysis of the second dimension of the rate-yield tradeoff. In (A) and (B), the growth rate of each ALE endpoint strain is labeled as black number beside the corresponding data point. (A) Two dimensions of the rate-yield tradeoff. The first dimension “d1” is the negative *μ*–*Y* correlation at maximum *Y*, and the second dimension “d2” is the negative *q*_*glc*_–*Y* correlation at a fixed *μ*. These correlations are observed in ME-model simulations and experimental data from ALE strains. (B) A correlation between *q*_*glc*_ and *q*_*ac*_ is also observed at fixed *μ* in both the ME-model and ALE endpoint data. Linear fits for the experimental data at similar growth rates are shown as dash-dotted (*μ* = 0.95–0.97 h^-1^), dashed (*μ* = 1.00–1.04 h^-1^), and solid (*μ* = 1.08–1.10 h^-1^) orange lines. These fits are described by the upper edges of the *q*_*glc*_–*q*_*ac*_ solution space at fixed *μ*. For growth between 1.00 and 1.04 h^-1^, *r*^2^ = 0.931 and *p* = 0.035. For growth between 1.08 and 1.1 h^-1^, *r*^2^ = 0.986 and *p* = 0.071. (C) The reaction fluxes in ME-model simulations along the upper edge (maximizing *q*_*ac*_) of the solution space for *μ* = 1.05 h^-1^ in (B). Notably, the P/O ratio (ratio of ATPS flux and oxygen uptake flux, gray dashed curve) is decreasing with increasing *q*_*glc*_. (D) Variation of fluxes distribution under the same *μ* in central metabolism and electron transport chain. (E) Model simulation: the ac-CoA split fraction to TCA cycle (CS) and acetate fermentation (PTAr). Here, two growth rates (0.95 *h*^−1^ and 1.05 *h*^−1^) are picked for simulation. (F) Experimental verification of the ac-CoA split through ^13^C-MFA data, details of the ^13^C-MFA data are illustrated in “2 ^13^C metabolic flux analysis” in S1 Appendix. **Abbreviations:CS: Citrate synthase** (***gltA***); **PTAr: Phosphotransacetylase** (***pta*** and ***eutD***); **ac**: acetate excretion; **THD2pp**: NAD(P) transhydrogenase (catalyzed by the gene product of *pntAB*); **NADH16pp**: NADH dehydrogenase (*nuoA–N*); **NADH5**: NADH dehydrogenase (*ndh*; **GND**: Phosphogluconate dehydrogenase (*gnd*); **GAPD**: Glyceraldehyde-3-phosphate dehydrogenase (*gapA*); **ATPS**: ATP synthase (*atpA–I*).

To see the relationship between the three variables *μ*, *q*_*glc*_, and *Y* we generated ME-model solution spaces in *q*_*glc*_ and *Y* at increasing lower bounds of *μ* (Fig 3A). These solution spaces represent the flexibility in the model to achieve a particular growth rate. At the *Y*-maximized limit of these solution space, we see the established negative *μ*–*Y* tradeoff where increasing growth rate requires increasing *q*_*glc*_ and decreasing *Y* (dashed arrow marked as “d1” in Fig 3A) coupling with increasing *q*_*ac*_ (top edges of solution spaces in Fig 3B). This is the first dimension of the rate-yield tradeoff, “d1”.

Considering only “d1”, one would expect the acetate production rate in all strains to be fully defined by the growth rate. In the case of this 1-dimensional tradeoff, all points in Fig 3A would appear on the line at the top of the blue solution spaces (parallel to the dotted “d1” line). However, we observed another degree of freedom in the phenotypic space. At a given *μ*, evolved strains can acquire higher *q*_*glc*_, higher *q*_*ac*_, and lower *Y*. The “d2” tradeoff is defined by a linear correlation in *q*_*glc*_–*q*_*ac*_ (Fig 3B) and a corresponding inverse proportional tradeoff in *q*_*glc*_–*Y* (Fig 3A).

The “d2” tradeoff is also predicted by ME-model simulations. At a given *μ*, the ME-model solution spaces extend toward lower *Y* and higher *q*_*glc*_, revealing this inverse proportional relationship in *q*_*glc*_-*Y*. The second dimension “d2” can also be seen in *q*_*ac*_–*q*_*glc*_ where the ME-model predicts the *q*_*ac*_–*q*_*glc*_ correlation observed in ALE endpoint as the *q*_*ac*_–maximized edges of the solution spaces (Fig 3B). The solution spaces predicted by ME-model show broad feasible ranges of acetate production *q*_*ac*_ at a given *q*_*glc*_ and *μ* (“bold” solution spaces in Fig 3B), so the *q*_*glc*_–*Y* tradeoff is not required by the model. On the other hand, the relationship between *q*_*glc*_ and *Y* is a strict tradeoff in the model (“thin” solution spaces in Fig 3A). Therefore, the ME-model suggests that *q*_*glc*_–*Y* is the more fundamental second dimension of the rate-yield tradeoff. To verify that hypothesis, one would look for mutant strains where *q*_*glc*_ increased while the other three phenotypic variables remained fixed (a shift to the right in Fig 3B).

### Mechanisms for the additional rate-yield tradeoff

We sought to identify the particular alternate metabolic strategies in the ME-model that could enable a *q*_*glc*_–*Y* tradeoff by identifying the differential pathway usage at a fixed high *μ* (1.05 h^-1^ in the ME-model (Fig 3C). The model predicts that when *q*_*glc*_ increases from the *Y*-maximized state (minimum *q*_*glc*_), flux through the proton-coupled NAD(P) transhydrogenase increases (reaction THD2pp, catalyzed by *pntAB*. In addition, a pathway switch between two different NADH dehydrogenase reactions, NADH5 (*ndh* and NADH16pp (*nuo*, appears at high *q*_*glc*_. In fact, each of or any combination of the 24 reactions in S6 Table can be activated in the ME-model to achieve high *q*_*glc*_, high *q*_*ac*_, and low *Y*. There are two common threads among these pathway activations. First, they all reduce the P/O ratio in the simulations (Fig 3C). NADH5 transports fewer protons to the periplasm per electron than NADH16pp. And increasing THD2pp flux drains the proton gradient without contributing to ATP production, thereby reducing P/O ratio (Fig 3D). Second, with the activation of those 24 reactions, glycolytic flux increases (Fig 3D) and pentose phosphate pathway flux decreases (Fig 3C). By comparing to the ^13^C metabolic flux analysis (Fig 3E and 3F), the ME-model shows quantitative predictive power for the second-order rate-yield tradeoff.

**Fig 4:**
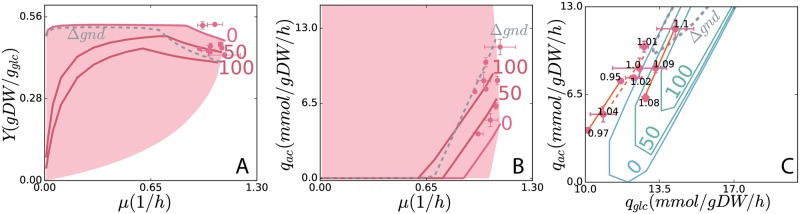
The second-order rate-yield tradeoff demonstrated by decreasing the P/O ratio and and knocking out *gnd* (“Δ*gnd*”) in ME-model simulations. The drop of P/O ratio is achieved by inducing the proton leakage (“PL”, a pseudo reaction of proton leakage added in the ME-model, details in “Materials and methods.”) reaction flux, as 0, 50 mmol gDW^-1^ h^-1^ (labeled “50”), and 100 mmol gDW^-1^ h^-1^ (labeled “100”). The new *Y*-maximized solution curves (solid red for “PL” flux variation, dashed grey for Δ*gnd*) in the (A) *μ*–*Y* and (B) *μ*–*q*_*ac*_ solution spaces. (C) The *q*_*ac*_–*q*_*glc*_ solution space contours (fixed *μ* = 1.0 h^-1^, solid blue for “PL” flux variation, dashed grey for Δ*gnd*) were simulated in the ME-model. Growth rates of the experimental strains are labeled as black numbers right next to each data point. The data points in similarly closed growth rates are connected by orange lines.

Experiments that introduce proton leakage have shown a shift towards high *q*_*ac*_ and low *Y* [5] in the same *μ*. It has also been shown that the variation of P/O ratio can uncouple the regulation of cytochrome oxidase from the cellular ATP demand [4]. More broadly, energy dissipation through proton leakage is known to be a method of metabolic control in bacteria [28, 29]. To clarify the effect of decreasing of P/O ratio in the ME-model, we added a reaction in the model representing proton leakage (Methods). As a result, we see the *Y*-maximized solution with decreased P/O ratios have higher *q*_*glc*_, higher *q*_*ac*_, and lower *Y* at a given *μ* (Fig 4). Finally, experiments have shown that knocking out *gnd* leads to increased *q*_*glc*_ and *q*_*ac*_ and decreased *Y* with little change in *μ* [30]. The ME-model also predicts that *gnd* knockout mutants (“gnd knockout simulation” Methods) will have increased *q*_*glc*_, *q*_*ac*_ and decreased *Y* (Fig 4). Since the ALE experiments do not introduce leaky proton or knock out any genes, it is also possible that multiple mechanisms working together, where the ME-model points to the systemic mechanisms for this fundamental second-order tradeoff. The exact pathways involved can be determined in future experiments.

Alternative explanations of the rate-yield tradeoff have been proposed, including membrane [31, 32] and cytosolic crowding [33, 34]. It is difficult to rule out these alternative constraints on cell growth, and it may be that multiple constraints operate at the same time. However, it is encouraging to see that the ME-model can explain the complex relationship between *μ*, *Y*, *q*_*ac*_, and *q*_*glc*_ with only metabolic and proteome allocation constraints. In the future, it will be possible to extend ME-models with additional constraints. For example, it has been proposed that the unmodeled protein fraction(UPF) is growth-rate dependent, and thus existing proteome allocation models with fixed UPF are inaccurate [34]. If this is indeed the case, then SSME- and ME-models with cytosolic crowding constraints can be developed to fully represent the interplay between crowding, proteome allocation, and pathway selection.

## Discussion

The *E. coli* ME-model provides a mechanistic and predictive model of rate-yield tradeoffs. It successfully reconciles several experimental data sets: i) uptake titration at low growth [8], ii) batch culture at higher growth rates [5], and iii) ALE endpoint strains (this study). These data sets, when analyzed with the ME-model, show the existence of a two-dimensional rate-yield tradeoff. The first dimension (“d1”) rate-yield tradeoff is *μ*–*Y* tradeoff and the second dimension (“d2”) is *q*_*glc*_–*Y* tradeoff.

From a mathematical perspective, one can describe the observed tradeoffs as correlations between any pair of the four variables *μ*, *Y*, *q*_*glc*_, and *q*_*ac*_. The two particular dimensions of the tradeoff that we describe, *μ*–*Y* (“d1”) and *q*_*glc*_–*Y* (“d2”), are motivated by two different trends in our physiological observations. First, the previously-reported strong linear correlation between *μ* and *Y* [5] occurs for isogenic cultures under carbon limitation. The second dimension “d2” appears when comparing laboratory evolution endpoint strains, where *q*_*glc*_, *q*_*ac*_, and *Y* are observed to vary at a fixed *μ*, with a linear relationship in *q*_*glc*_–*q*_*ac*_ and a corresponding inverse proportional relationship in *q*_*glc*_–*Y*. This two-dimensional tradeoff cannot be deciphered from simpler intuitive models, but it can be derived from the comprehensive set of metabolic and gene expression pathways represented by the ME-model.

Furthermore, this study employed a multi-scale modeling approach where a small-scale model was used to guide parameter estimation in the genome-scale ME-model. This approach—which has been termed Tunable Resolution (TR) modeling [35]—was essential to the success of the study, and we expect that both small-scale and genome-scale models will continue to play an important role in understanding the genotype-phenotype relationship.

The two-dimensional rate-yield tradeoff appears as a result of ALE selection for *μ* when alternative pathway selection strategies achieve the same growth rate. Proton leakage and alternative ETC pathway selection are plausible mechanisms for modifying the P/O ratio and creating the *q*_*glc*_–*Y* tradeoff. In addition, the flux ratio between glycolysis (GAPD, *gapA*) and the pentose phosphate pathway (GND, *gnd*) might play a significant role in the *q*_*glc*_–*Y* tradeoff. Those mechanisms can be tested experimentally. Finally, revealing the underlying regulation would be of great interest for establishing a deeper understanding of rate-yield tradeoffs. Combining ME-models with known regulatory mechanisms to explain cellular choices would achieve a long-standing goal in systems biology [36].

## Materials and methods

Phenotypic data including *μ*, *q*_*glc*_, *q*_*ac*_, and excretion rates of other metabolic byproducts were collected for ALE endpoint strains (“1 Phenotypic characterization of *E. coli* strains” in S1 Appendix). In addition, ^13^C fluxes were measured from two of the strains with different growth rate and different glucose yield (“2 ^13^C metabolic flux analysis” in S1 Appendix). Reference data points of rate-yield, growth-acetate relations of wild-type MG1655 and NCM3722 E. coli strains were collected from published studies [5, 8]. The coarse-grained proteome allocation model from [5] was reformulated as a small-scale ME-model (SSME-model, detail in “5 SSME-model parameters derivation” in S1 Appendix) and implemented by the COBRAme framework [16]. The genome-scale model *i*JL1678-ME was modified to fit experimental data by modifying the *k*_*eff*_s (enzyme turnover rate) of TCA cycle reactions, blocking target reactions, and modifying UPF (unmodeled protein fraction), GAM (growth associate maintenance energy), and NGAM (non-growth associate maintenance energy) (“8 Experimental data fitting” in S1 Appendix). Solution spaces were generated using flux balance analysis (incorporated in COBRAme) in the ME-model (“7 Solution space of the ME-model” in S1 Appendix). To determine the effect of modifying P/O ratio on ME-model solution spaces, a reaction representing proton leakage was added to the ME-model (“10 P/O ratio manipulation” in S1 Appendix). The effect of the *gnd* knockout was demonstrated by blocking the reaction GND in ME-model simulations (“11 gnd knockout simulation” in S1 Appendix).

## Supporting information

S1 Appendix Detailed introduction and discussions of materials and methods. (PDF)

S1 Table Parameters comparison between two coarse-grained models. Comparison between the coarse-grained proteome allocation model [5] and SSME-model. The derivation in detail is shown in “5 SSME-model parameter derivation” in S1 Appendix.
(PDF)

S2 Table ME-model parameters. Global parameter selection in iJL1678-ME model to fit the *μ*–*Y*, *μ*–*q*_*ac*_ data as in Fig 2C and 2D.
(PDF)

S3 Table iJL1678-ME model modification (blocked reactions). Reactions that need to be turned off in the model to get quantitative fit of *μ*–*Y*, *μ*–*q*_*ac*_ data as in Fig 2C and 2D.
(XLSX)

S4 Table Essential exchanges. Boundary reactions in the ME-model that need to be turned on.
(XLSX)

S5 Table Solution space variation (Below). Reactions that after being turned off, *q*_*ac*,*min*_ increases. The variation of *μ*–*q*_*ac*_ solution space is shown in S6B Fig.
(XLSX)

S6 Table Solution space variation (Above). Reactions that after being turned off, *q*_*ac*,*max*_ decreases. The *μ*–*q*_*ac*_ solution space would varied as shown in S6A Fig.
(XLSX)

S7 Table ALE phenotypes measurements. *μ*, *q*_*ac*_ and *q*_*glc*_ measurements of the *E. coli* adapted MG1655 strains. Strains are replicates from [3].
(XLSX)

S8 Table *E. coli* K-12 MG1655 WT phenotypes measurements. *μ*, *q*_*ac*_ and *q*_*glc*_ measurements. Data from [3].
(XLSX)

S9 Table *E. coli* NCM3722 glucose uptake titration phenotypes. Data from [5].
(XLSX)

S10 Table *E. coli* K-12 MG1655 chemostat measurements. Data from [8].
(XLSX)

S11 Table ^13^C metabolic flux analysis data. Metabolic fluxes distribution of the highest *μ* strain and highest *Y* strain among the ALE endpoint strains.
Tab “Reactions” ^13^C MFA model and carbon mapping network.Tab “Net_fluxes” ^13^C MFA calculated net fluxes. LB and UB are the 95% confidence intervals.Tab “SymMets” ^13^C MFA model symmetric metabolite carbon mappings.Tab “MS_data” Measured mass distribution vectors (MDVs) by LC-MS/MS and their associated carbon mappings used for MFA calculations.Tab “Flux_data” Measured uptake and secretion rates by HPLC.
(XLSX)

S1 Fig Scheme of the coarse-grained proteome allocation model [5]. (TIF)

S2 Fig Modification of ME-model for fitting the experimental data, based on the guideline derived from SSME-model. (A) Reduction of the enzyme efficiency for respiration (*k*_*eff*,*res*_) causes a more gradual acetate line. Reduction of UPF increases the model-predicted maximum *μ*, shifting the acetate line to higher *μ*. (B) Another approach of getting more gradual acetate line is to block bp1 reactions. (C) Activation of bp2 reactions (such as the Entner–Doudoroff pathway bypassing glycolysis) cause an inflection point and extension of the acetate line to higher *μ*. (D) Workflow for the ME-model modification process. In the genome-scale ME-model, some TCA cycle reactions appeared as bp1 reactions, but, because they belong to the major respiration pathway of the cell, we will decreased their *k*_*eff*_s rather than blocking them entirely.
(TIF)

S3 Fig Summary of the modifications to the genome-scale ME-model. (A) Compared to original *i*JL1678-ME, unmodeled protein fraction (UPF) is halved to 18%. (B) For the enzyme efficiency parameter *k*_*eff*_, only the TCA *k*_*eff*_s are modified. (C) The subsystems of the 24 bp1 reactions. (D) The subsystems of 26 bp2 reactions. (E) bp1 and bp2 reactions on the pathway map of central metabolism. (F) Acetate lines for the steps in the fitting process. More detailed illustration process is shown in S4 Fig.
(TIF)

S4 Fig Iteration process of filling the ME-model prediction gap of growth rate dependent acetate excretion. First two steps of bp1 iteration process are shown in the left two figures, where as we block the first bp1 reaction (ICL), the slope (threshold) of the acetate line drops. The changes of the threshold (bp1 modification) and acetate line end point (bp2 modification) from iteration Step 3–21 are shown in the right figure. Step 3–18 are the modification on bp1 reactions, where the threshold (red squares) gradually drops from high growth to low growth. Step 19–21 are the modification on bp2 reactions, where the acetate line end point (in red circles) drops. The blue solid line is the final prediction of *μ*–*q*_*ac*_ relation, which is the same as the blue line in S3F Fig. More detail about bp1 and bp2 reactions are in S3 Table.
(TIF)

S5 Fig By blocking byproduct excretion pathways in the ME-model, which is verified by the experimental data, the solution space was reduced from the pink region to the yellow region. (TIF)

S6 Fig *μ*–*q*_*ac*_ solution space variation in the ME-model. Narrowing in the feasible range of alternative suboptimal solutions by blocking some target reactions. The new solution space after the variation is shown as the yellow in (A) and (B), with the original solution space in pink. (A) 24 target reactions (S6 Table) that are blocked where maximum *q*_*ac*_s in high *μ* get lower, where the upper edge of the yellow region is below the upper edge of the pink region. The activation of one of these 24 reactions thus corresponding to higher *q*_*ac*_ with lower *Y*. (B) 11 target reactions (S5 Table) corresponding to lower *q*_*ac*_ with lower Y, blocking those reactions will get the minimum *q*_*ac*_ (lower edge of the yellow region) closed to the *Y*-maximized *q*_*ac*_ solution. (C) The method of picking reactions to block: Looking for the reactions that are not activated in the yield-maximized solution but activated at the maximal and minimal of the *μ*–*q*_*ac*_ solution space, where the principal is to keep the *Y*-maximized solutions unchanged.
(TIF)

S7 Fig Expansion of solution space from the SSME-model by adding model reactions. The expanded part of the solution space is shown as yellow in (A)–(C), compared to the original SSME-model solution spaces are in blue. (A) All added reactions ((1)-(4) in D) expand the solution space to include lower-*Y* solutions (B) Reactions (1) and (3) expand the solution space to low-*q*_*ac*_ at high *μ*. (C) Reactions (2) and (4) expand the solution space to high-*q*_*ac*_ across all *μ*. (D) Model reactions that are added in the SSME-model for expanding the original solution space, all those reactions are guaranteed not be activated in the Y-maximized solutions so that the *Y*-optimal solution remains the same to fit data from [5]. Reaction (1) corresponds to the reactions that would generate products other than acetate such as pyruvate excretion, lactate excretion, etc. Reaction (2) is representative to the reactions that would generate other products, but at the same time generating acetate, such as pyruvate formate lyase (PFL), which produce formate and acetyl-CoA (precursor of acetate) from pyruvate. Reaction (3) and (4) could both be referred from the futile cycle in energy production and consumption, where (3) are the reactions that are less efficient than the optimal pathway, such as the alternative reactions in ETC which are less efficient in transporting electrons, while (4) are the reactions that would waste more energy in the same growth comparing to the optimal state, such as the reactions that would cause proton leakage.
(TIF)
