# Supplementary material for: Laboratory evolution reveals a two-dimensional rate-yield tradeoff in microbial metabolism
Source: PLoS Comput Biol. 2019 Jun 3;15(6):e1007066. doi: 10.1371/journal.pcbi.1007066 (PMC6564042; doi:10.1371/journal.pcbi.1007066)
Supplement: S1 Appendix — (PDF) [file pcbi.1007066.s001.pdf]

S1 APPENDIX

Appendix: Laboratory evolution reveals a two-dimensional rate-yield tradeoff in microbial metabolism

Chuankai Cheng[1] | Edward J. O'Brien[1] | Douglas McCloskey[1] | Jose Utrilla[1] | Connor Olson[1] | Ryan A. LaCroix[1] | Troy E. Sandberg[1] | Adam M. Feist[1,2] | Bernhard O. Palsson[1,2,3] | Zachary A. King[1]

[1]Department of Bioengineering, University of California San Diego, La Jolla, CA 92093, USA

[2]Novo Nordisk Foundation Center for Biosustainability, Technical University of Denmark, 2800 Lyngby, Denmark.

[3]Department of Pediatrics, University of California San Diego, La Jolla, CA 92093, USA

Correspondence
Zachary A. King, Department of Bioengineering, University of California San Diego, La Jolla, CA 92093, USA
Email: zaking@ucsd.edu

Funding information
Funding for this research was provided by the Novo Nordisk Foundation through the Center for Biosustainability at the Technical University of Denmark (NNF10CC1016517).

This is the appendix for "Laboratory evolution reveals a two-dimensional rate-yield tradeoff in microbial metabolism", by Cheng et al.

CONTENTS

1 Phenotypic characterization of *E. coli* strains 2

2 ^{13}C metabolic flux analysis 3

3 Proteome constraints in the ME-model 3

4 Connection between proteome allocation model and ME-model 4

5 SSME-model parameter derivation 4

6 ME-model Matlab and COBRAme Implementation 8

7 Solution space of the ME-model 8

8 Experimental data fitting 9

9 Solution space variation 10

10 P/O ratio manipulation 11

11 *gnd* knockout simulation 11

12 Tables 11

13 Figures 14

14 References 15

1 | PHENOTYPIC CHARACTERIZATION OF *E. COLI* STRAINS

Characterization of adaptive laboratory evolution (ALE) clones from LaCroix et al. (2015) was performed using clonal isolate stocks which were inoculated into overnight cultures, and then passaged twice in late exponential phase after approximately 5–6 generations in order to characterize cells which were physiologically adapted to the growth conditions. The media used was identical to the M9 minimal media with 4 g/L glucose as described (LaCroix et al., 2015) and were fully oxygenated in flasks with approximately 15 mL working volume. Optical density measurements at a 600 nm wavelength were taken periodically with a spectrophotometer (Thermo Fisher Scientific, Waltham, MA) until stationary phase was reached, and were correlated to dry weight. At each sample point, the cell culture was filtered through a 0.22 um PVDF membrane (MilliporeSigma, Burlington, MA), and the filtrate was collected. By-products and substrates were quantified in the filtrate by high-performance liquid chromatography using a refractive-index detector (Agilent Technologies, Santa Clara, CA) and an Aminex HPX-87H column (Bio-Rad Laboratories, Hercules, CA). Uptake rates were calculated using the slope of a best linear fit of the concentration of an analyte over the dry cell weight, multiplied by the growth rate over the same exponential growth region.

2 | ^{13}C METABOLIC FLUX ANALYSIS

Two of the ALE strains, index 7B(strain with highest growth yield) and index 10 (strain with lowest growth yield) in Table S11 have been collected for ^{13}C metabolic flux analysis (MFA). Triplicate cultures were grown on labeled glucose M9 minimal media (Sambrook and Russell, 2001) with trace elements (Fong et al., 2005) and sampled from a heat block that was maintained at 37 °C and fully aerated with tumble stir magnets. The labeled tracer consisted of 20/80 mixture of ^{13}C glucose and 1-^{13}C glucose purchased from Cambridge Isotope Laboratories, Inc. (Tewksbury, MA). Samples were taken and extracted using a modified version of the fast Swinnex filtration approach described previously (McCloskey et al., 2015b).

A XSELECT HSS XP 150 mm × 2.1 mm × 2.5 µm (Waters®, Milford, MA) with a Prominence UFLC XR HPLC (Shimadzu, Columbia, MD) was used for chromatographic separation (McCloskey et al., 2015a). An AB SCIEX Qtrap 5500 mass spectrometer (AB SCIEX, Framingham, MA) operated in negative mode with was used for detection of metabolites (McCloskey et al., 2015a). Precursor and product isotopomers of selected metabolites were calculated from multiple-reaction monitoring. Additional product isotopomers were calculated from enhanced product ion (EPI) scans coupled to an information dependent acquisition (IDA) method. Details of the isotopomer calculations and acquisition method can be referred to McCloskey et al. (2016a,b).

A core model derived from the central carbohydrate metabolism and biomass composition of iJO1366 (Orth et al., 2014) was utilized for MFA simulations to confirm the succinate dehydrogenase knockout and confirm elimination of flux from succinate to fumarate. A core model of *E. coli* central metabolism based on the model by Leighty and Antoniewicz (2012) with slight modifications to include an alternative glucose importer and an additional reaction to include the metabolite Phosphoribosyl pyrophosphate (prpp) was utilized for MFA simulations involving the glucose evolved endpoint strains. Substrate uptake and secretion rates calculated from triplicate cultures grown on 12C glucose M9 minimal media were used as additional constraints. MFA simulations were conducted using MATLAB and INCA v1.3 (Young, 2014). Confidence intervals were calculated using a method similar to that described in Antoniewicz et al. (2006) as encoded in INCA (Young, 2014). Standard deviations were calculated based off of 95% confidence intervals as described in Antoniewicz et al. (2006).

3 | PROTEOME CONSTRAINTS IN THE ME-MODEL

The specific growth rate μ is the number of replicated cell per time unit per cell. When the cell replicates, it needs to replicate all cellular components proportionally, and this can be represented as a steady-state dilution rate $q_{dilution}$ of each individual component C (Molenaar et al., 2009).

$$q_{dilution,C} = \mu\,[C]\,,\, C \in cell\ components \tag{1}$$

In Eq. 1, $[C]$ is the concentration of the components in the original cell.

Beginning with Michaellis-Menten kinetics defined by the equation:

$$q_{reaction} = \frac{k_{cat}[E][S]}{K_M + [S]} \tag{2}$$

We can derive a bulk parameter k_{eff} where:

$$k_{eff} = \frac{k_{cat}[S]}{K_M + [S]} \tag{3}$$

$$q_{reaction} = k_{eff}[E] \tag{4}$$

The ME-model formulation is based on the inequality form of Eq. 4 (Lloyd et al., 2018; O'brien et al., 2013):

$$q_{reaction} \leq k_{eff,E} \cdot [E] = k_{eff,E} \cdot \frac{q_{dilution,E}}{\mu} \tag{5}$$

4 | CONNECTION BETWEEN PROTEOME ALLOCATION MODEL AND ME-MODEL

The proteome allocation model presented by (Basan et al., 2015) defined proteome efficiency with the symbol ε as the proportionality coefficient relating reaction rate through a metabolic pathway, q_r, to the proteome fraction, ϕ. ϕ represents the fraction of total cell proteome dedicated to catalyzing flux through the pathway.

$$q_r = \varepsilon \cdot \phi_E \tag{6}$$

Next, they assume that the size of the entire proteome is linearly dependent on the growth rate μ with the coefficient γ. For a particular enzyme E, the dilution rate $q_{dilution,E}$ must provide enough enzyme to catalyze flux, thus:

$$\phi_{E,reaction} \leq \frac{q_{dilution,E}}{\gamma\mu} \tag{7}$$

Combining with Eq. 5, we have:

$$k_{eff,E} = \varepsilon_E/\gamma \tag{8}$$

Thus, a simple model assigning parameters from the coarse-grained proteome allocation model (Basan et al., 2015) to the ME-model can be built. We call it small scale ME-model (SSME-model).

5 | SSME-MODEL PARAMETER DERIVATION

The SSME-model is derived from the course-grained proteome allocation model presented by (Basan et al., 2015) (Fig. S1). The model contains three pathways, respiration (shown as "res"), fermentation ("fer"), and biomass synthe-

sis pathway ("bms"). The respiration pathway generates energy (ATP) to feed the synthesis of biomass and excrete CO2. The fermentation pathway generates energy and excrete acetate. The biomass pathway synthesizes biomass proportionally to the growth rate μ with proportionality coefficient β.

According to Basan et al. (2015), the coupling constraint on each pathway is based on proteome allocation which can be explained in three steps (Fig.S1):

- The entire biomass ($\beta\mu$) can be separated into proteome with the coefficient α and other biomass with the coefficient $1 - \alpha$ (including lipids, nucleotides, and other components). This is based on the assumption that the total fraction of proteome in the cell is unchanged; data suggests the actual variation is within 5% (Mori et al., 2016).
- The proteome is separated into the proteins for the respiration, fermentation, and biomass pathways with the coefficient ϕ_{max} and the proteome for other pathways, $1 - \phi_{max}$.
- The proteome is finally divided into the protein for each pathway with coefficients ϕ_r for respiration, ϕ_f for fermentation, and $1 - \phi_r - \phi_f$ for biomass.

Fluxes through the respiration and fermentation pathways are notated as $q_{E,r}$ and $q_{E,f}$. The total energy that is created through these two pathways must meet the energy demand for growth:

$$q_{E,f} + q_{E,r} = q_E(\mu) = \sigma\mu \tag{9}$$

The flux of carbon substrate uptake is annotated as $q_{C,in}$, and the fluxes that consuming substrate through the respiration and fermentation pathways are $q_{C,f}$ and $q_{C,r}$. The rest of the carbon substrate contributes directly to biomass production, $q_{C,bms}$:

$$q_{C,bms}(\mu) = q_{C,in} - q_{C,f} - q_{C,r} = \beta\mu \tag{10}$$

The growth energy flux and biomass carbon flux could be considered as the demand reaction that their reaction rates are:

$$\begin{cases} q_{demand,energy} = q_E(\mu) = \sigma\mu \\ q_{demand,biomass} = q_{C,bms}(\mu) = \beta\mu \end{cases} \tag{11}$$

The substrate uptake rate bound (SURB) is considered to be equivalent to the maximum value of the carbon input flux: $q_{C,in} \leq SURB$. For the respiration and fermentation pathways, the rates are normalized to the carbon flux of the two pathways. The amount of energy that is created through either respiration or fermentation is proportional to the carbon flux with coefficients e_r and e_f:

$$\begin{cases} q_r = q_{C,r} = q_{E,r}/e_r \\ q_f = q_{C,f} = q_{E,f}/e_f \end{cases} \tag{12}$$

Assuming that the proteome possesses a fixed portion α ($0 \leq \alpha \leq 1$) of the biomass content:

$$\begin{cases} v_{demand,proteome} = \alpha \cdot v_{demand,biomass}(\mu) = \alpha \cdot \beta\mu \\ v_{demand,otherbiomass} = (1-\alpha) \cdot \beta\mu \end{cases} \tag{13}$$

The proteome is divided into the growth-dependent proteome $\mu DPro$ with the maximum fraction ϕ_{max} ($0 \leq \phi_{max} \leq 1$) and growth-independent proteome $\mu IPro$:

$$\begin{cases} q_{demand,\mu DPro} = \phi_{max} \cdot q_{demand,proteome} = \phi_{max} \cdot \alpha \cdot \beta\mu \\ q_{demand,\mu IPro} = (1-\phi_{max} \cdot \alpha \cdot \beta\mu \end{cases} \tag{14}$$

The growth-dependent proteome is divided into three parts corresponding the respiration, fermentation, and biomass pathways.

$$\phi_f + \phi_r + \phi_{BM} = 1 \tag{15}$$

Where the proteome part for the biomass would be divided into two parts, a fixed portion annotated by ϕ_0 and a portion whose amount is linearly related to the growth by coefficient b.

$$\phi_{BM} = \phi_0 + b\mu \tag{16}$$

Therefore, the entire proteome demand is:

$$\begin{aligned} & q_{demand,proteome} \\ &= q_{demand,\mu DPro} + q_{demand,\mu IPro} \\ &= (\phi_f + \phi_r + \phi_0 + b\mu) \cdot \phi_{max} \cdot \alpha \cdot \beta\mu + (1-\phi_{max} \cdot \alpha \cdot \beta\mu \end{aligned} \tag{17}$$

Thus, for the fixed portion ϕ_0 of the growth dependent-proteome, the dilution rate is:

$$q_{demand,proteome_0} = \phi_0 \cdot \phi_{max} \cdot \alpha \cdot \beta\mu \tag{18}$$

And, for the growth-independent proteome:

$$q_{demand,proteome_0} = (1-\phi_{max} \cdot \alpha \cdot \beta\mu) \tag{19}$$

The portions of the proteome catalyzing respiration and fermentation pathways are proportional to the energy

flux.

$$\begin{cases} \phi_r = \frac{q_{E,r}}{\epsilon_r} = \frac{e_r \cdot q_r}{\epsilon_r} \\ \phi_f = \frac{q_{E,f}}{\epsilon_f} = \frac{e_f \cdot q_f}{\epsilon_f} \end{cases} \tag{20}$$

The demand for cellular components required for growth is less than or equal to the dilution fluxes that represent their production rate (in the same way that Eq. 5 was derived).

$$q_{demand,E} \leq q_{dilution,E}, (E \in catalytic\ components) \tag{21}$$

Consequently, for the dilution rate of the proteome catalyzing the respiration pathway:

$$\begin{aligned} & q_{dilution,proteome_r} \\ & \geq q_{demand,proteome} \cdot \phi_{max} \cdot \phi_r \\ & = \alpha \cdot \beta\mu \cdot \phi_{max} \cdot \frac{e_r \cdot q_r}{\epsilon_r} \end{aligned} \tag{22}$$

And therefore:

$$\begin{aligned} q_r &\leq \frac{\epsilon_r}{e_r \cdot \alpha \cdot \beta \cdot \phi_{max}} \cdot \frac{q_{dilution,proteome_r}}{\mu} \\ &= k_{eff,r} \times \frac{q_{dilution,proteome_r}}{\mu} \end{aligned} \tag{23}$$

Equivalently for the fermentation pathway:

$$\begin{aligned} q_f &\leq \frac{\epsilon_f}{e_f \cdot \alpha \cdot \beta \cdot \phi_{max}} \cdot \frac{q_{dilution,proteome_f}}{\mu} \\ &= k_{eff,f} \times \frac{q_{dilution,proteome_f}}{\mu} \end{aligned} \tag{24}$$

And lastly for the biomass pathway:

$$\begin{aligned} & q_{dilution,proteome_bms} \\ & \geq q_{demand,proteome} \cdot \phi_{max} \cdot (b\mu) \end{aligned} \tag{25}$$

$$\begin{aligned} q_{demand,proteome} &\leq \frac{1}{\phi_{max} \cdot b} \cdot \frac{q_{dilution,proteome_{bms}}}{\mu} \\ &= k_{eff,proteome_{bms}} \times \frac{q_{dilution,proteome_{bms}}}{\mu} \end{aligned} \tag{26}$$

These are equivalent to the inequalities in the ME-model (Eq. 5), and thus were able to implement the SSME-Model in COBRAme.

$$a_{demand,energy} = \sigma\mu + w \tag{27}$$

$$\begin{aligned} & q_{demand,LacZ} \\ &= q_{demand,proteome} \cdot \phi_Z \\ &= \alpha \cdot \beta\mu \cdot \phi_Z \end{aligned} \tag{28}$$

$$q_{demand,proteome_0} = \phi_0 \cdot (\phi_{max} - \phi_Z) \tag{29}$$

6 | ME-MODEL MATLAB AND COBRAME IMPLEMENTATION

This study provided the SSME-model implemented in Matlab (2017a) with the non-linear solver (“fmincon” function, https://www.mathworks.com/help/optim/ug/fmincon.html), as a general example of how ME-model is formulated and simulated.

The genome-scale ME-model iJL1678-ME is gained from Lloyd et al. (2018), which is available on the ecolime github repository (https://github.com/SBRG/ECOLIme), implemented using Python(2.7/3.6). For the solver, both SoPlex and Qminos (Yang et al., 2016; Ma et al., 2017) have been tested and getting same solutions in this study.

A github repository has been created (https://github.com/ahoiching/ME-model-rate-yield-tradeoff) for the details of the implementation process.

7 | SOLUTION SPACE OF THE ME-MODEL

The COBRAme framework (Lloyd et al., 2018) was used for simulations of the *E. coli* ME-model *i*JL1678-ME (Liu et al., 2014) and the SSME-model. All fermentation product excretions except acetate were disabled in *i*JL1678-ME to match the experimental observation that only acetate is excreted during aerobic growth in ALE-optimized strains and reported strains (Basan et al., 2015; Nanchen et al., 2006). Table S4 in Expanded view shows all the essential excretion components (including acetate) that are not blocked. Additional modifications to the ME-model are described below (see "Experimental data fitting").

Solution spaces in two dimensions were generated using flux balance analysis (FBA). First, the independent variable (a reaction flux) was maximized and minimized using FBA to establish the range of feasible values. Next, for intervals across this range, the independent variable was fixed and the dependent variable was maximized and minimized using FBA.

With this approach, we were able to define the solution spaces for μ–Y, μ-q_{ac}, and q_{glc}-q_{ac} in the ME-model. To generate μ–Y and μ–q_{ac} solution spaces, the ME-model was optimized for the objectives of Y_{max}, Y_{min}, $q_{ac,max}$, and $q_{ac,min}$ at each feasible μ. To determine the feasible range of μ, μ was to maximized with the carbon uptake (q_{glc})

unbounded, and the range was thus between 0 (no growth) and μ_{max}. Y_{min} was calculated by minimized q_{glc} at each feasible μ, while Y_{min} was calculated by maximizing q_{glc}.

The q_{glc}–q_{ac} solution space shows the feasible range of q_{ac} at each feasible q_{glc} when μ is fixed. According to the μ–Y solution space, the feasible range of q_{glc} at each μ can be calculated to generate $q_{glc,min}$ and $q_{glc,max}$. The acetate overflow rate q_{ac} was then maximized and minimized across this range.

8 | EXPERIMENTAL DATA FITTING

The linear-threshold response of acetate overflow (q_{ac}) upon growth (μ) is a key phenotype to reproduce in the ME-model. Due to the poor quantitative prediction of μ–Y and μ–q_{ac} from *i*JL1678-ME (Lloyd et al., 2018; O'brien et al., 2013) with the default parameter set—the linear threshold response μ-q_{ac} is too steep compared to experimental data—modification to the model was required. Because the rate-yield tradeoff is determined by a tradeoff between metabolic efficiency and proteome efficiency, the following parameters in *i*JL1678-ME were modified: enzyme turnover rates (k_{eff}) of the reactions within the TCA cycle, upper bound and lower bound constraints for certain target reactions (described below), unmodeled protein fraction (UPF), non-growth-associated maintenance (NGAM), and growth-associated maintenance (GAM).

The k_{eff} modifications are based on protein abundance data (Basan et al., 2015), where the k_{eff} of each reaction in the TCA cycle is replaced by the measured protein efficiencies. The measured protein efficiencies turned out to be lower than the originally assigned TCA k_{eff}s in *i*JL1678-ME.

The upper bound and lower bound constraints are used to block some reactions by setting both of them to 0 mmol gDW^{-1} h^{-1}. In order to fit the linear-threshold response q_{ac} from experiments, two kinds of backup pathways ("bp") were identified. The first set of "bp" reactions (categorized as bp1) have direct negative correlations to acetate overflow as a function of μ (Fig. S2B). These are alternative pathways whose metabolic and proteomic efficiencies are in between the acetate production and TCA pathways. Fig. S2B shows that when a bp1 reaction is blocked, the acetate production line becomes more gradual. With an iteration process, each reaction can be blocked, then another bp1 reaction becomes activates in the next simulation, this reaction can be blocked, and so forth. As a result, 17 steps of bp1 iteration were employed to match the slope of the measure acetate line (Basan et al., 2015) (Fig. S3F, Table S3 from).

Another set of "bp" reactions (categorized as bp2) have direct positive correlations with μ but are only activated in near-optimal μ (Fig. S2C). Blocking bp2 reactions helps maintain the linearity of the acetate production response in the ME-model. As a result, four steps of bp2 iteration were executed (Table S3 from Expanded view).

The rationale for blocking bp1 and bp2 reactions was carefully considered. On the computational side, blocking reactions enabled an accurate fit to experimental data (Fig. S3F). More importantly, for a given set of k_{eff}s, the iteration process generates a minimal set of reactions that must be blocked to fit the acetate line. For example, Table S3 shows that ICL is the first bp1 reaction that is blocked. Even if the other 16 bp1 reactions are blocked, ICL prevents the model from achieving a good fit to the data.

Blocking these reactions is the most straightforward approach to constraining them, but a similar effects could be achieved by: (A) adding other upper bound and lower bound except for zero, reflecting the possibility that those reactions are restricted not to carry high fluxes, (B) lowering the k_{eff}s, reflecting that those reactions might carry higher protein costs, (C) changing the coefficients of the metabolites, making it less metabolic efficient, for example to reflect the requirement of cofactors. Taking these approach needs more thorough consideration based on biochemical evidence.

By exploring the literature, we were able to categorize and understand some bp1 and bp2 (Fig. S3C-E): (A) short-

cuts within the TCA cycle, (B) alternative pathways of the glycolysis/electron transport chain, (C) lipid and cell wall/ envelope biosynthesis, or (D) catabolism of amino acids and nucleotides feeding for energy synthesis. Among all those reactions, some reactions are measured to carry no flux in higher growth by chemostats glucose uptake experiments, such as ED, ICL, and ASPT (Nanchen et al., 2006; Novak et al., 2006); some are likely to have low k_{eff} in higher growth, such as the reactions in the ETC or lipid membrane synthesis, since the cells in fast growth has less membrane availability, inducing the protein cost (Zhuang et al., 2011; Szenk et al., 2017); some of the reactions are predicted to be blocked being caused by some autoregulation mechanism, requiring a cofactor with high protein cost, such as FTHFD, requiring methionine, the amino acid that cost the most protein to get synthesized (Meinnel et al., 1993; Nagy et al., 1995), or in a simpler explanation, the k_{eff} is too high in the original ME-model. Some other rationales of the bp1 blockage would be hard to be determined but could raise up an interesting topic in cellular regulations study. For instance, the 4th bp1 reaction that is found, NAD transhydrogenase (NADTRHD), reducing NAD while oxidizing NADP, might need to be blocked to create a similar effect when the NAD/NADP balance is regulated. There are some researches focusing on the NAD/NADP balance (Osterman, 2009; Auriol et al., 2011), but how would this affect the acetate overflow is not thoroughly studied yet. More details about the effect of each reaction to the overall metabolic pathway when it is turned on is recorded in Table S3 (Expanded view).

For the global parameters, unmodeled protein fraction (UPF) provides global effects to the model as it shifts the acetate overflow line in parallel left or right, which means changing the maximum growth while maintaining the same phenotype depending on growth.

The result of the modification process is shown in Fig. S3F. The original *i*JL1678-ME model predicts little acetate production around μ_{max} (0.81 h^{-1}. After the modification of TCA k_{eff}s, q_{ac} at μ_{max} approximately matches measured data reported by Basan et al. (2015), but the μ–q_{ac} is still too steep. By modifying the UPF, the μ_{max} increases to 1.03 h^{-1}. By blocking the bp1 reactions step by step, the slope of μ–q_{ac} line becomes more gradual and finally fits the experimental data well. By blocking the bp2 reactions, $q_{ac,max}$ becomes closer to the maximum acetate overflow in experiments.

The detailed iteration process of bp1 and bp2 modification is shown in Fig. S4. As we are blocking bp1 in the iterative sequence, the slope (threshold) of the acetate line gradually decreases. And blocking the bp2 reaction leads to the drop of the end point of the acetate line. We believe that this is the best combination of bp1 and bp2 modification because in every iteration step, only one or one set of bp1 or bp2 reaction can be identified, which means there is no other choices. We assumes that the iteration process results in the best fit is because in the last few steps of iteration, the change of the acetate line becomes tiny, and finally, no bp1 or bp2 can be further identified. In another words, the iteration converges.

The other global parameters, NGAM and GAM, were manipulated to fit the level of Y to data. Specifically, NGAM captures the positive μ–Y correlation at low μ and GAM varies the maximum value of Y. The results of picking these parameters are shown in Table S2, allowing us to fit 3 sets of experimental data (Fig. 2C–D in the main text).

9 | SOLUTION SPACE VARIATION

The SSME-model and ME-model share a common theoretical basis, so it is possible to gradually add content to the SSME-model until it becomes equivalent to the genome-scale ME-model. We defined a method for modifying the solution spaces of the SSME-model and ME-model to explore what model components are responsible for the difference between the solution spaces generated by the SSME-model and the genome-scale ME-model. We aimed to determine the reactions in the genome-scale model that could narrow the solution space if blocked and the reactions that could

be added to the SSME-model to expand the solution space.

The principal of this method is to find reactions whose addition or removal do not affect the linear-threshold response of acetate production, which is also the growth-yield-maximized solution. Thus, we compared the metabolic fluxes between the growth-yield-optimized solution and the acetate-maximized or -minimized solution, then blocked the reaction that carried flux in the maximized or minimized solution but not in the growth-yield-optimized solution. As shown in Fig. S6, this is an iteration process, a new target reaction appeared to be activated only when the preceding reaction is blocked and a new solution is simulated. Reactions that are found in the iteration process are then categorized as 4 kinds with different properties, and testified by adding model reactions in the SSME-model and see how the SSME-model solution space expands (Fig. S7). As for the ALE measurements show no other fermentation products besides acetate excreted (category (1) in Fig. S7D), the unnecessary exchange reactions are then blocked in the model (Table S4 shows the essential exchange reactions), where the ME-model solution space got smaller (yellow solution space in Fig. S5). The yellow solution spaces shown in Fig. S5 is the same as the solution spaces encompassing ALE data points in the main text (Fig. 2 in main text).

10 | P/O RATIO MANIPULATION

To simulate the modification of the P/O ratio in the ME-model, a pseudo-reaction for proton leakage was added to the model that pumped one proton from the periplasm to the cytosol. For simulations in the manuscript, flux through this reaction was fixed to 50 or 100 mmol gDW^{-1} h^{-1}.

The P/O ratio is calculated by the ratio between the ATP synthase flux and the oxygen uptake flux.

11 | *GND* KNOCKOUT SIMULATION

To simulate Δ*gnd*, the metabolic reaction phosphogluconate dehydrogenase (GND) in the ME-model was blocked by setting the upper and lower bounds to 0 mmol gDW^{-1} h^{-1}.

12 | TABLES

TABLE S1 **Parameters comparison between two coarse-grained models.** Comparison between the coarse-grained proteome allocation model Basan et al. (2015) and SSME-model. The derivation in detail is shown in "5 SSME-model parameter derivation".

Basan's	Value	SSME-model	Value	Comments
ϕ_0	81%	UPF	81%	Same concept
σ	45.7(mM/OD)	σ Energy + β Carbon substrate −> Biomass	45.7 Energy + 28.5 Carbon substrate −>1.0 Biomass	
β	28.5(mM/OD)			
S_{ac}	1/3	Carbon substrate −>S_{ac} Acetate + e_f Energy	1.0 Carbon substrate −>1/3 Acetate + 2.0 Energy	Incorporate as reaction stoichiometry in SSME-model
e_f	2.0			
S_{CO_2}	1/6	Carbon substrate−> S_{CO_2} CO_2 + e_r Energy	1.0 Carbon substrate−> 1/6 CO_2 + 4.4 Energy	
e_r	4.4			
b	12.0%	$k_{eff,bms} = 1/b$	8.33/3600 (mM/OD/sec)	Incorporated as k_{eff} values in SSME-model where the time unit is second
ε_f	750(mM/OD/hr)	$k_{eff,fer} = \varepsilon_f/e_f$	375/3600 (mM/OD/sec)	
ε_r	390(mM/OD/hr)	$k_{eff,res} = \varepsilon_r/e_r$	88.6364/3600 (mM/OD/sec)	

ϕ_0, umodeled protein fraction (UPF)

$\sigma(\beta)$, energy (carbon) demand for growth

$S_{ac}(S_{CO_2}$, stoichiometry factor for acetate (CO_2) from fermentation (respiration)

$e_f(e_r)$, carbon efficiency, fermentation (respiration)

$\varepsilon_f(\varepsilon_r)$, proteome efficiency, fermentation (respiration)

b, proteomic sector for supporting biomass reaction

TABLE S2 **ME-model parameters.** Global parameter selection in iJL1678-ME model to fit the μ–Y, μ–q_{ac} data as in Fig. 2C and D.

Model Scale	Experiment	UPF (%)	GAM (mmol/gDW/hr)	NGAM (mmol/gDW/hr)
Small scale	(Nanchen et al., 2006)	0.82	75	0
	(Basan et al., 2015)	0.81	45.7	0
	ALE	0.80	40	0
Genome scale	(Nanchen et al., 2006)	0.30	34.98	15
	(Basan et al., 2015)	0.18	34.98	1
	ALE	0.12	15	0

UPF, unmodeled protein fraction

(N)GAM, (non)growth associated maintenance for energy

TABLE S3 **iJL1678-ME model modification (blocked reactions).** Reactions that need to be turned off in the model to get quantitative fit of μ–Y, μ–q_{ac} data as in Fig. 2C and D.

TABLE S4 **Essential exchanges.** Boundary reactions in the ME-model that need to be turned on.

TABLE S5 **Solution space variation (Below).** Reactions that after being turned off, $q_{ac,min}$ increases. The variation of μ–q_{ac} solution space is shown in Fig. S6B.

TABLE S6 **Solution space variation (Above).** Reactions that after being turned off, $q_{ac,max}$ decreases. The μ–q_{ac} solution space would varied as shown in Table S6A.

TABLE S7 **ALE phenotypes measurements.** μ, q_{ac} and q_{glc} measurements of the *E. coli* adapted MG1655 strains. Strains are replicates from LaCroix et al. (2015).

TABLE S8 ***E. coli* K-12 MG1655 WT phenotypes measurements.** μ, q_{ac} and q_{glc} measurements. Data from LaCroix et al. (2015).

TABLE S9 ***E. coli* NCM3722 glucose uptake titration phenotypes.** Data from Basan et al. (2015).

TABLE S10 ***E. coli* K-12 MG1655 chemostat measurements.** Data from Nanchen et al. (2006).

TABLE S11 **^{13}C metabolic flux analysis data.** Metabolic fluxes distribution of the highest μ strain and highest Y strain among the ALE endpoint strains.

- Tab "Reactions" ^{13}C MFA model and carbon mapping network.
- Tab "Net_fluxes" ^{13}C MFA calculated net fluxes. LB and UB are the 95% confidence intervals.
- Tab "SymMets" ^{13}C MFA model symmetric metabolite carbon mappings.
- Tab "MS_data" Measured mass distribution vectors (MDVs) by LC-MS/MS and their associated carbon mappings used for MFA calculations.
- Tab "Flux_data" Measured uptake and secretion rates by HPLC.

13 | FIGURES

FIGURE S1 **Scheme of the coarse-grained proteome allocation model Basan et al. (2015).**

FIGURE S2 **Modification of ME-model for fitting the experimental data, based on the guideline derived from SSME-model.** (A) Reduction of the enzyme efficiency for respiration ($k_{eff,res}$) causes a more gradual acetate line. Reduction of UPF increases the model-predicted maximum μ, shifting the acetate line to higher μ. (B) Another approach of getting more gradual acetate line is to block bp1 reactions. (C) Activation of bp2 reactions (such as the Entner–Doudoroff pathway bypassing glycolysis) cause an inflection point and extension of the acetate line to higher μ. (D) Workflow for the ME-model modification process. In the genome-scale ME-model, some TCA cycle reactions appeared as bp1 reactions, but, because they belong to the major respiration pathway of the cell, we will decreased their k_{eff}s rather than blocking them entirely.

FIGURE S3 **Summary of the modifications to the genome-scale ME-model.** (A) Compared to original *i*JL1678-ME, unmodeled protein fraction (UPF) is halved to 18%. (B) For the enzyme efficiency parameter k_{eff}, only the TCA k_{eff}s are modified. (C) The subsystems of the 24 bp1 reactions. (D) The subsystems of 26 bp2 reactions. (E) bp1 and bp2 reactions on the pathway map of central metabolism. (F) Acetate lines for the steps in the fitting process. More detailed illustration process is shown in Fig. S4.

FIGURE S4 **Iteration process of filling the ME-model prediction gap of growth rate dependent acetate excretion.** First two steps of bp1 iteration process are shown in the left two figures, where as we block the first bp1 reaction (ICL), the slope (threshold) of the acetate line drops. The changes of the threshold (bp1 modification) and acetate line end point (bp2 modification) from iteration Step 3–21 are shown in the right figure. Step 3–18 are the modification on bp1 reactions, where the threshold (red squares) gradually drops from high growth to low growth. Step 19–21 are the modification on bp2 reactions, where the acetate line end point (in red circles) drops. The blue solid line is the final prediction of μ–q_{ac} relation, which is the same as the blue line in S3–F. More detail about bp1 and bp2 reactions are in Table S3.

FIGURE S5 **By blocking byproduct excretion pathways in the ME-model, which is verified by the experimental data, the solution space was reduced from the pink region to the yellow region.**

FIGURE S6 **μ–q_{ac} solution space variation in the ME-model.** Narrowing in the feasible range of alternative suboptimal solutions by blocking some target reactions. The new solution space after the variation is shown as the yellow in (A) and (B), with the original solution space in pink. (A) 24 target reactions (Table S6) that are blocked where maximum q_{ac}s in high μ get lower, where the upper edge of the yellow region is below the upper edge of the pink region. The activation of one of these 24 reactions thus corresponding to higher q_{ac} with lower Y. (B) 11 target reactions (Table S5) corresponding to lower q_{ac} with lower Y, blocking those reactions will get the minimum q_{ac} (lower edge of the yellow region) closed to the Y-maximized q_{ac} solution. (C) The method of picking reactions to block: Looking for the reactions that are not activated in the yield-maximized solution but activated at the maximal and minimal of the μ–q_{ac}solution space, where the principal is to keep the Y-maximized solutions unchanged.

FIGURE S7 **Expansion of solution space from the SSME-model by adding model reactions.** The expanded part of the solution space is shown as yellow in (A)–(C), compared to the original SSME-model solution spaces are in blue. (A) All added reactions ((1)-(4) in D) expand the solution space to include lower-Y solutions (B) Reactions (1) and (3) expand the solution space to low-q_{ac} at high μ. (C) Reactions (2) and (4) expand the solution space to high-q_{ac} across all μ. (D) Model reactions that are added in the SSME-model for expanding the original solution space, all those reactions are guaranteed not be activated in the Y-maximized solutions so that the Y-optimal solution remains the same to fit data from Basan et al. (2015). Reaction (1) corresponds to the reactions that would generate products other than acetate such as pyruvate excretion, lactate excretion, etc. Reaction (2) is representative to the reactions that would generate other products, but at the same time generating acetate, such as pyruvate formate lyase (PFL), which produce formate and acetyl-CoA (precursor of acetate) from pyruvate. Reaction (3) and (4) could both be referred from the futile cycle in energy production and consumption, where (3) are the reactions that are less efficient than the optimal pathway, such as the alternative reactions in ETC which are less efficient in transporting electrons, while (4) are the reactions that would waste more energy in the same growth comparing to the optimal state, such as the reactions that would cause proton leakage.

14 | REFERENCES

REFERENCES

Antoniewicz, M. R., Kelleher, J. K. and Stephanopoulos, G. (2006) Determination of confidence intervals of metabolic fluxes estimated from stable isotope measurements. *Metab. Eng.*, **8**, 324–337.

Auriol, C., Bestel-Corre, G., Claude, J.-B., Soucaille, P. and Meynial-Salles, I. (2011) Stress-induced evolution of escherichia coli points to original concepts in respiratory cofactor selectivity. *Proc. Natl. Acad. Sci. U. S. A.*, **108**, 1278–1283.

Basan, M., Hui, S., Okano, H., Zhang, Z., Shen, Y., Williamson, J. R. and Hwa, T. (2015) Overflow metabolism in escherichia coli results from efficient proteome allocation. *Nature*, **528**, 99–104.

Fong, S. S., Burgard, A. P., Herring, C. D., Knight, E. M., Blattner, F. R., Maranas, C. D. and Palsson, B. O. (2005) In silico design and adaptive evolution of escherichia coli for production of lactic acid. *Biotechnol. Bioeng.*, **91**, 643–648.

LaCroix, R. A., Sandberg, T. E., O'Brien, E. J., Utrilla, J., Ebrahim, A., Guzman, G. I., Szubin, R., Palsson, B. O. and Feist, A. M. (2015) Use of adaptive laboratory evolution to discover key mutations enabling rapid growth of escherichia coli K-12 MG1655 on glucose minimal medium. *Appl. Environ. Microbiol.*, **81**, 17–30.

Leighty, R. W. and Antoniewicz, M. R. (2012) Parallel labeling experiments with [U-13C]glucose validate e. coli metabolic network model for 13C metabolic flux analysis. *Metab. Eng.*, **14**, 533–541.

Liu, J. K., O'Brien, E. J., Lerman, J. A., Zengler, K., Palsson, B. O. and Feist, A. M. (2014) Reconstruction and modeling protein translocation and compartmentalization in escherichia coli at the genome-scale. *BMC Syst. Biol.*, **8**, 110.

Lloyd, C. J., Ebrahim, A., Yang, L., King, Z. A., Catoiu, E., O'Brien, E. J., Liu, J. K. and Palsson, B. O. (2018) COBRAme: A computational framework for genome-scale models of metabolism and gene expression. *PLoS Comput. Biol.*, **14**, e1006302.

Ma, D., Yang, L., Fleming, R. M. T., Thiele, I., Palsson, B. O. and Saunders, M. A. (2017) Reliable and efficient solution of genome-scale models of metabolism and macromolecular expression. *Sci. Rep.*, **7**, 40863.

McCloskey, D., Gangoiti, J. A., Palsson, B. O. and Feist, A. M. (2015a) A ph and solvent optimized reverse-phase ion-paring-LC–MS/MS method that leverages multiple scan-types for targeted absolute quantification of intracellular metabolites. *Metabolomics*, **11**, 1338–1350.

McCloskey, D., Utrilla, J., Naviaux, R. K., Palsson, B. O. and Feist, A. M. (2015b) Fast swinnex filtration (FSF): a fast and robust sampling and extraction method suitable for metabolomics analysis of cultures grown in complex media. *Metabolomics*, **11**, 198–209.

McCloskey, D., Young, J. D., Xu, S., Palsson, B. O. and Feist, A. M. (2016a) MID max: LC-MS/MS method for measuring the precursor and product mass isotopomer distributions of metabolic intermediates and cofactors for metabolic flux analysis applications. *Anal. Chem.*, **88**, 1362–1370.

— (2016b) Modeling method for increased precision and scope of directly measurable fluxes at a Genome-Scale. *Anal. Chem.*, **88**, 3844–3852.

Meinnel, T., Mechulam, Y. and Blanquet, S. (1993) Methionine as translation start signal: a review of the enzymes of the pathway in escherichia coli. *Biochimie*, **75**, 1061–1075.

Molenaar, D., van Berlo, R., de Ridder, D. and Teusink, B. (2009) Shifts in growth strategies reflect tradeoffs in cellular economics. *Mol. Syst. Biol.*, **5**, 323.

Mori, M., Hwa, T., Martin, O. C., De Martino, A. and Marinari, E. (2016) Constrained allocation flux balance analysis. *PLoS Comput. Biol.*, **12**, e1004913.

Nagy, P. L., Marolewski, A., Benkovic, S. J. and Zalkin, H. (1995) Formyltetrahydrofolate hydrolase, a regulatory enzyme that functions to balance pools of tetrahydrofolate and one-carbon tetrahydrofolate adducts in escherichia coli. *J. Bacteriol.*, **177**, 1292–1298.

Nanchen, A., Schicker, A. and Sauer, U. (2006) Nonlinear dependency of intracellular fluxes on growth rate in miniaturized continuous cultures of escherichia coli. *Appl. Environ. Microbiol.*, **72**, 1164–1172.

Novak, M., Pfeiffer, T., Lenski, R. E., Sauer, U. and Bonhoeffer, S. (2006) Experimental tests for an evolutionary trade-off between growth rate and yield in e. coli. *Am. Nat.*, **168**, 242–251.

O'brien, E. J., Lerman, J. A., Chang, R. L., Hyduke, D. R. and Palsson, B. Ø. (2013) Genome-scale models of metabolism and gene expression extend and refine growth phenotype prediction. *Mol. Syst. Biol.*, **9**, 693.

Orth, J. D., Conrad, T. M., Na, J., Lerman, J. A., Nam, H., Feist, A. M. and Palsson, B. O. (2014) A comprehensive genome-scale reconstruction of escherichia coli metabolism–2011. *Mol. Syst. Biol.*, **7**, 535–535.

Osterman, A. (2009) Biogenesis and homeostasis of nicotinamide adenine dinucleotide cofactor. *EcoSal Plus*, **3**.

Sambrook, J. and Russell, D. W. (2001) Molecular cloning: a laboratory manual., 3rd edn.(cold spring harbor laboratory press: Cold spring harbor, new york).

Szenk, M., Dill, K. A. and de Graff, A. M. R. (2017) Why do Fast-Growing bacteria enter overflow metabolism? testing the membrane real estate hypothesis. *Cell Syst*, **5**, 95–104.

Yang, L., Ma, D., Ebrahim, A., Lloyd, C. J., Saunders, M. A. and Palsson, B. O. (2016) solveME: fast and reliable solution of nonlinear ME models. *BMC Bioinformatics*, **17**, 391.

Young, J. D. (2014) INCA: a computational platform for isotopically non-stationary metabolic flux analysis. *Bioinformatics*, **30**, 1333–1335.

Zhuang, K., Vemuri, G. N. and Mahadevan, R. (2011) Economics of membrane occupancy and respiro-fermentation. *Mol. Syst. Biol.*, **7**, 500.
