# Supplementary material for: Laboratory evolution reveals a two-dimensional rate-yield tradeoff in microbial metabolism
Source: PLoS Comput Biol. 2019 Jun 3;15(6):e1007066. doi: 10.1371/journal.pcbi.1007066 (PMC6564042; doi:10.1371/journal.pcbi.1007066)
Supplement: S1 Table — Comparison between the coarse-grained proteome allocation model [5] and SSME-model. The derivation in detail is shown in “5 SSME-model parameter derivation” in S1 Appendix. (PDF) [file pcbi.1007066.s002.pdf]

TABLE S1 Comparison between the coarse-grained proteome allocation model (Basan et al., 2015) and SSME-model

Basan's	Value	SSME-model	Value	Comments
ϕ_0	81%	UPF	81%	Same concept
σ<br>β	45.7(mM/OD)<br>28.5(mM/OD)	σ Energy<br>+ β Carbon substrate<br>–> Biomass	45.7 Energy<br>+ 28.5 Carbon substrate<br>–>1.0 Biomass	
S_{ac}<br>e_f	1/3<br>2.0	Carbon substrate<br>–>S_{ac} Acetate<br>+ e_f Energy	1.0 Carbon substrate<br>–>1/3 Acetate<br>+ 2.0 Energy	Incorporate as reaction stoichiometry in SSME-model
S_{CO_2}<br>e_r	1/6<br>4.4	Carbon substrate–><br>S_{CO_2} CO_2<br>+ e_r Energy	1.0 Carbon substrate–><br>1/6 CO_2<br>+ 4.4 Energy	
b	12.0%	$k_{eff,bms} = 1/b$	8.33/3600<br>(mM/OD/sec)	Incorporated as k_{eff} values in SSME-model where the time unit is second
ε_f	750(mM/OD/hr)	$k_{eff,fer} = \varepsilon_f / e_f$	375/3600<br>(mM/OD/sec)	
ε_r	390(mM/OD/hr)	$k_{eff,res} = \varepsilon_r / e_r$	88.6364/3600<br>(mM/OD/sec)	

ϕ_0, umodeled protein fraction (UPF)
$\sigma(\beta)$, energy (carbon) demand for growth
$S_{ac}(S_{CO_2}$, stoichiometry factor for acetate (CO_2) from fermentation (respiration)
$e_f(e_r)$, carbon efficiency, fermentation (respiration)
$\varepsilon_f(\varepsilon_r)$, proteome efficiency, fermentation (respiration)
b, proteomic sector for supporting biomass reaction
