# Supplementary material for: Laboratory evolution reveals a two-dimensional rate-yield tradeoff in microbial metabolism
Source: PLoS Comput Biol. 2019 Jun 3;15(6):e1007066. doi: 10.1371/journal.pcbi.1007066 (PMC6564042; doi:10.1371/journal.pcbi.1007066)
Supplement: S2 Table — Global parameter selection in iJL1678-ME model to fit the μ–Y, μ–qac data as in Fig 2C and 2D. (PDF) [file pcbi.1007066.s003.pdf]

TABLE S2 Global parameter selection

Model Scale	Experiment	UPF (%)	GAM (mmol/gDW/hr)	NGAM (mmol/gDW/hr)
Small scale	(Nanchen et al., 2006)	0.82	75	0
	(Basan et al., 2015)	0.81	45.7	0
	ALE	0.80	40	0
Genome scale	(Nanchen et al., 2006)	0.30	34.98	15
	(Basan et al., 2015)	0.18	34.98	1
	ALE	0.12	15	0

UPF, unmodeled protein fraction

(N)GAM, (non)growth associated maintenance for energy
