# Supplementary figures and images for: Laboratory evolution reveals a two-dimensional rate-yield tradeoff in microbial metabolism
Source: PLoS Comput Biol. 2019 Jun 3;15(6):e1007066. doi: 10.1371/journal.pcbi.1007066 (PMC6564042; doi:10.1371/journal.pcbi.1007066)

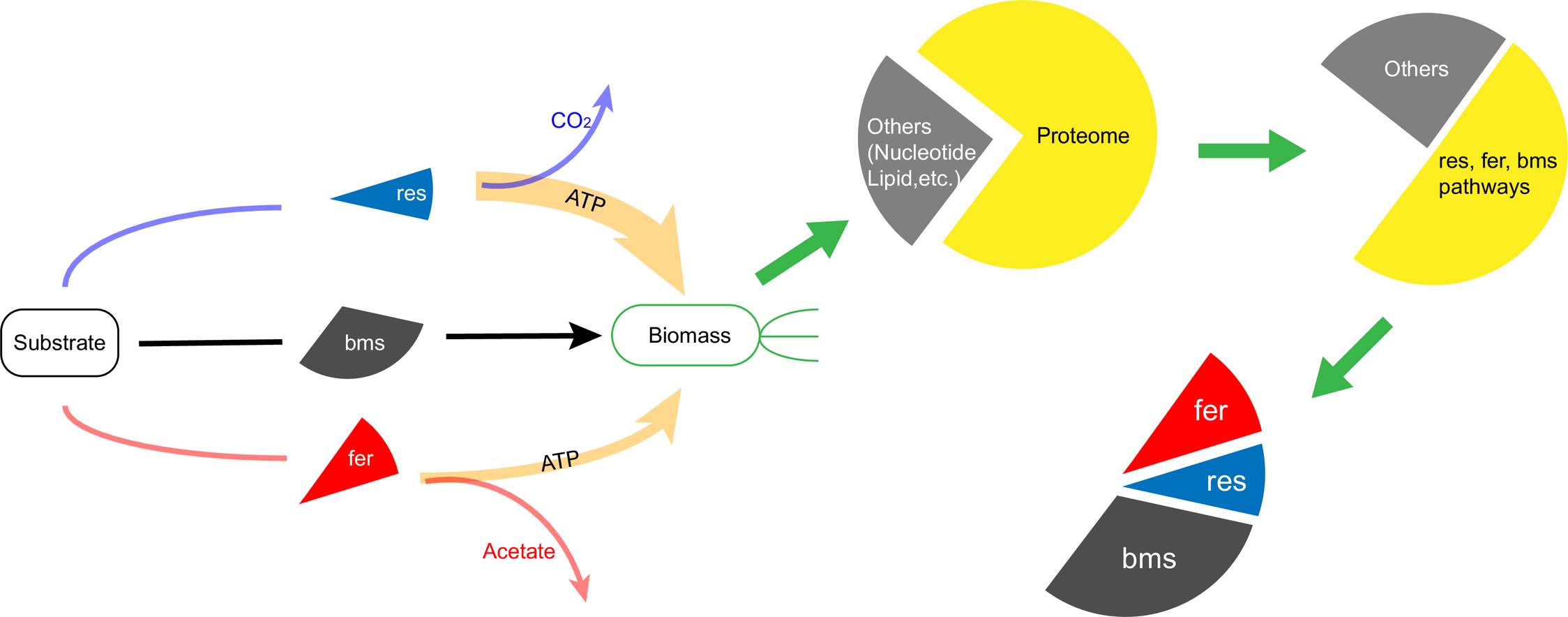

Supplement: S1 Fig — (TIF) [file pcbi.1007066.s013.tif]

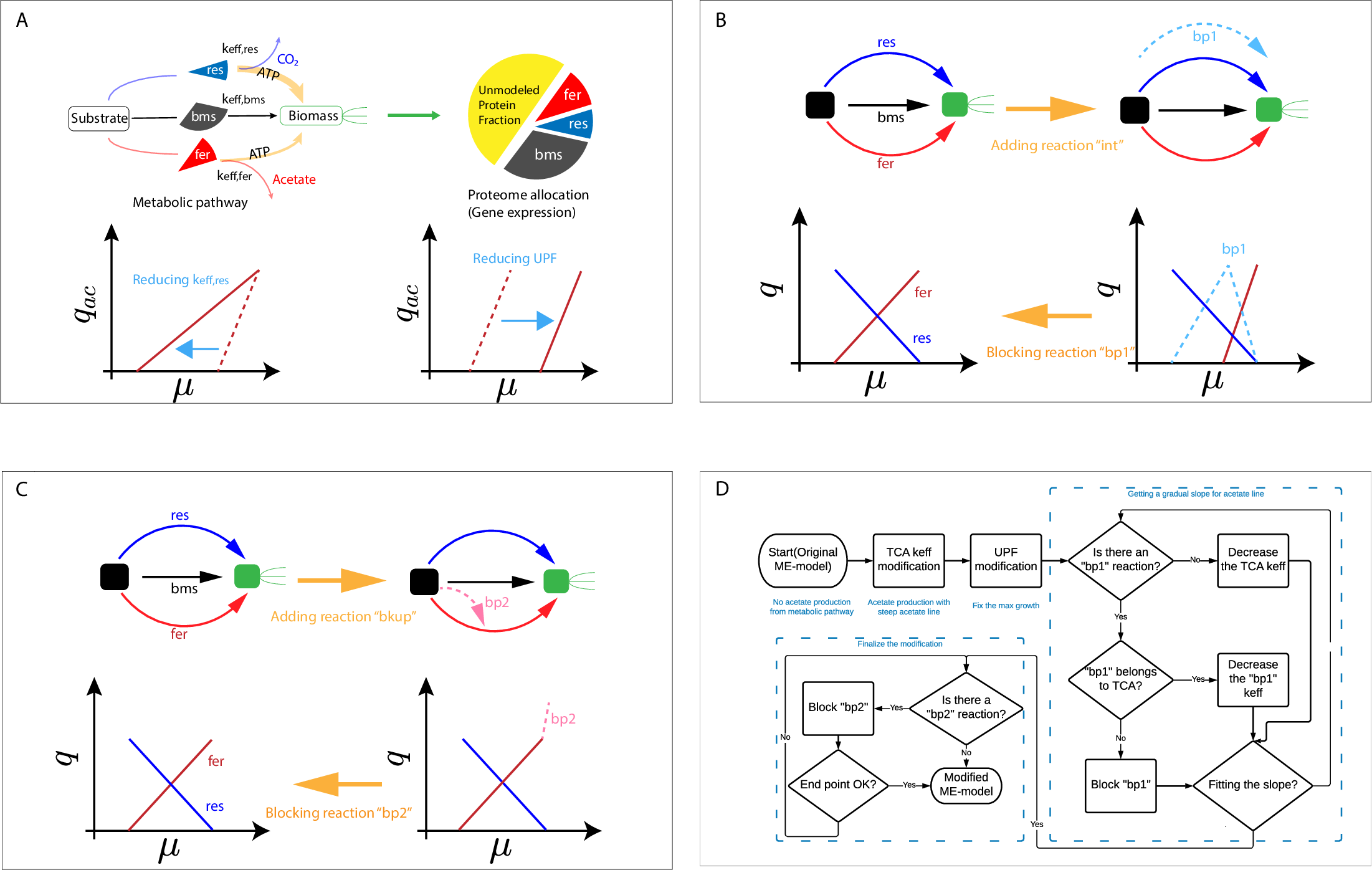

Supplement: S2 Fig — (A) Reduction of the enzyme efficiency for respiration (keff,res) causes a more gradual acetate line. Reduction of UPF increases the model-predicted maximum μ, shifting the acetate line to higher μ. (B) Another approach of getting more gradual acetate line is to block bp1 reactions. (C) Activation of bp2 reactions (such as the Entner–Doudoroff pathway bypassing glycolysis) cause an inflection point and extension of the acetate line to higher μ. (D) Workflow for the ME-model modification process. In the genome-scale ME-model, some TCA cycle reactions appeared as bp1 reactions, but, because they belong to the major respiration pathway of the cell, we will decreased their keffs rather than blocking them entirely. (TIF) [file pcbi.1007066.s014.tif]

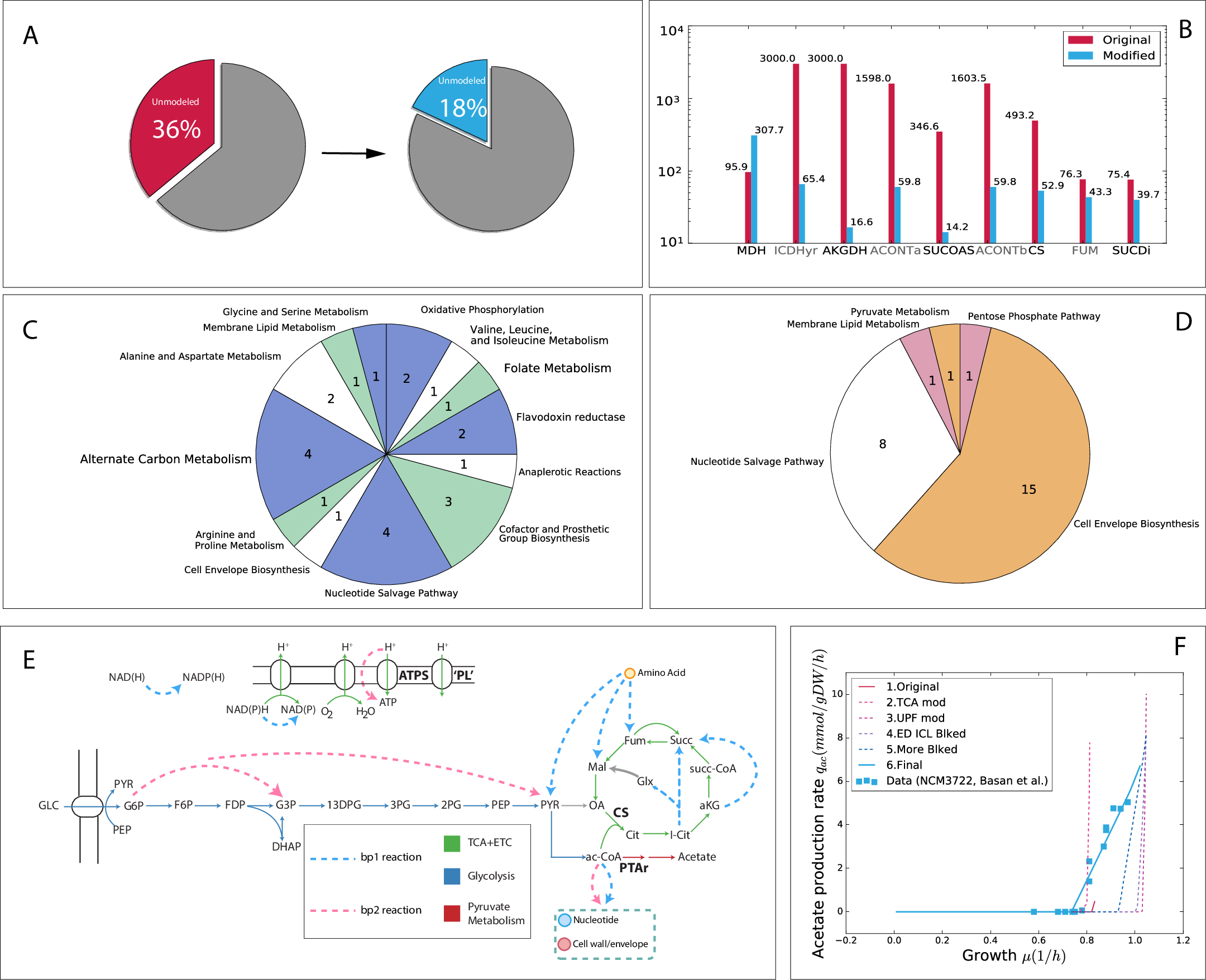

Supplement: S3 Fig — (A) Compared to original iJL1678-ME, unmodeled protein fraction (UPF) is halved to 18%. (B) For the enzyme efficiency parameter keff, only the TCA keffs are modified. (C) The subsystems of the 24 bp1 reactions. (D) The subsystems of 26 bp2 reactions. (E) bp1 and bp2 reactions on the pathway map of central metabolism. (F) Acetate lines for the steps in the fitting process. More detailed illustration process is shown in S4 Fig. (TIF) [file pcbi.1007066.s015.tif]

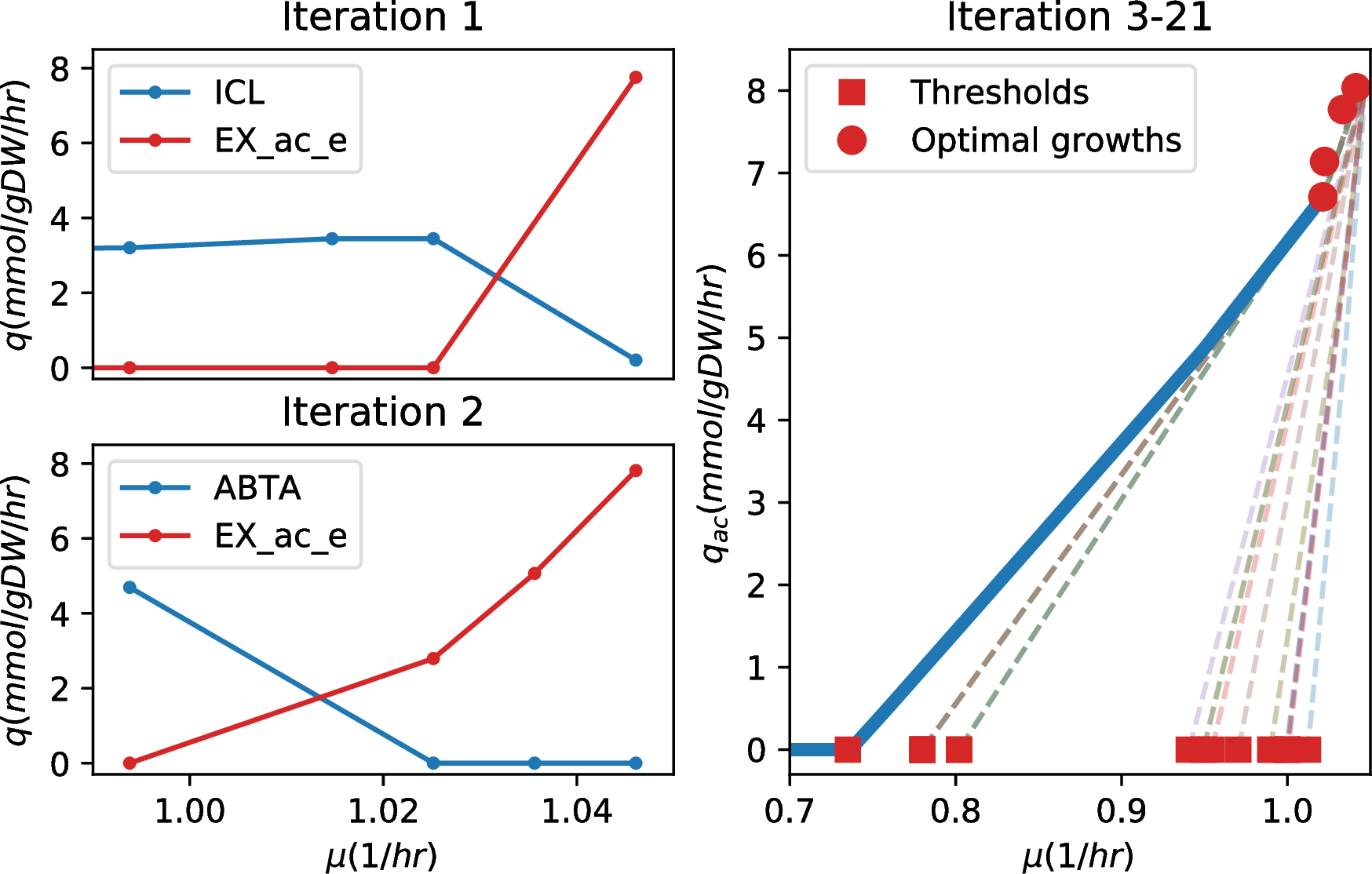

Supplement: S4 Fig — First two steps of bp1 iteration process are shown in the left two figures, where as we block the first bp1 reaction (ICL), the slope (threshold) of the acetate line drops. The changes of the threshold (bp1 modification) and acetate line end point (bp2 modification) from iteration Step 3–21 are shown in the right figure. Step 3–18 are the modification on bp1 reactions, where the threshold (red squares) gradually drops from high growth to low growth. Step 19–21 are the modification on bp2 reactions, where the acetate line end point (in red circles) drops. The blue solid line is the final prediction of μ–qac relation, which is the same as the blue line in S3F Fig. More detail about bp1 and bp2 reactions are in S3 Table. (TIF) [file pcbi.1007066.s016.tif]

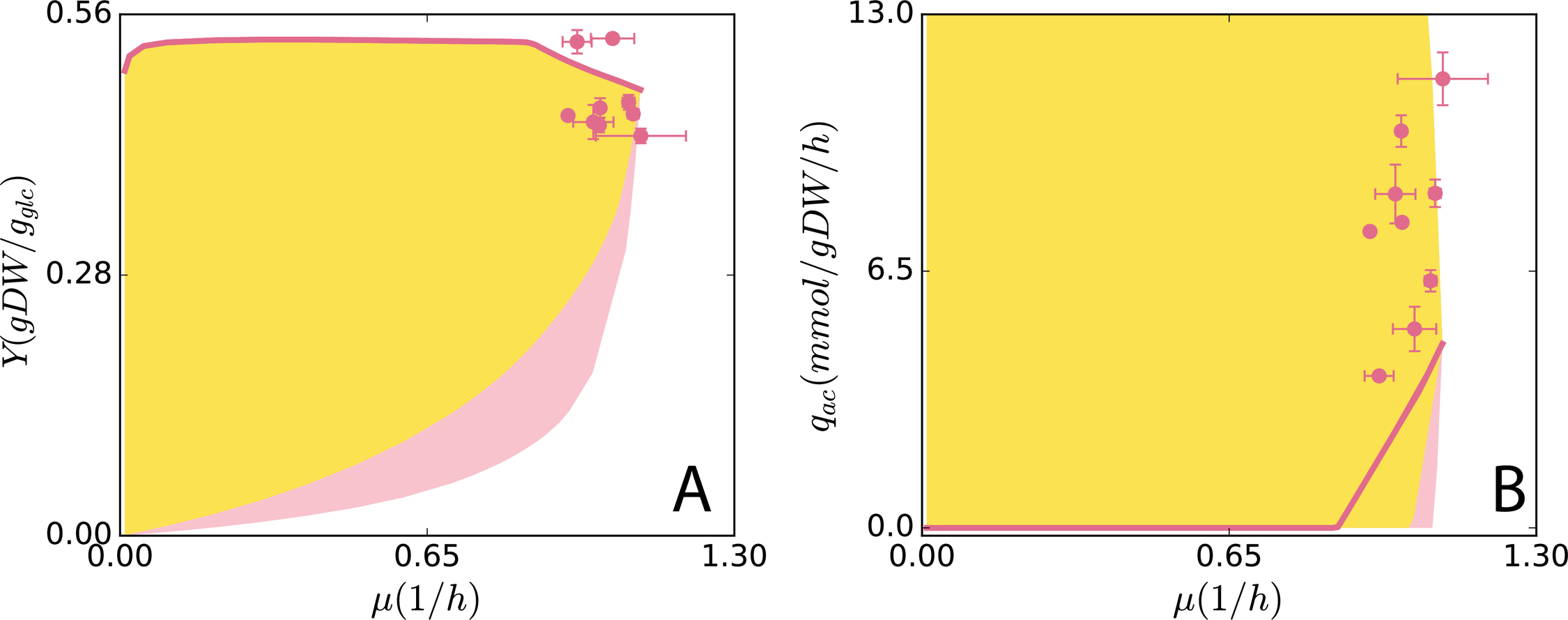

Supplement: S5 Fig — (TIF) [file pcbi.1007066.s017.tif]

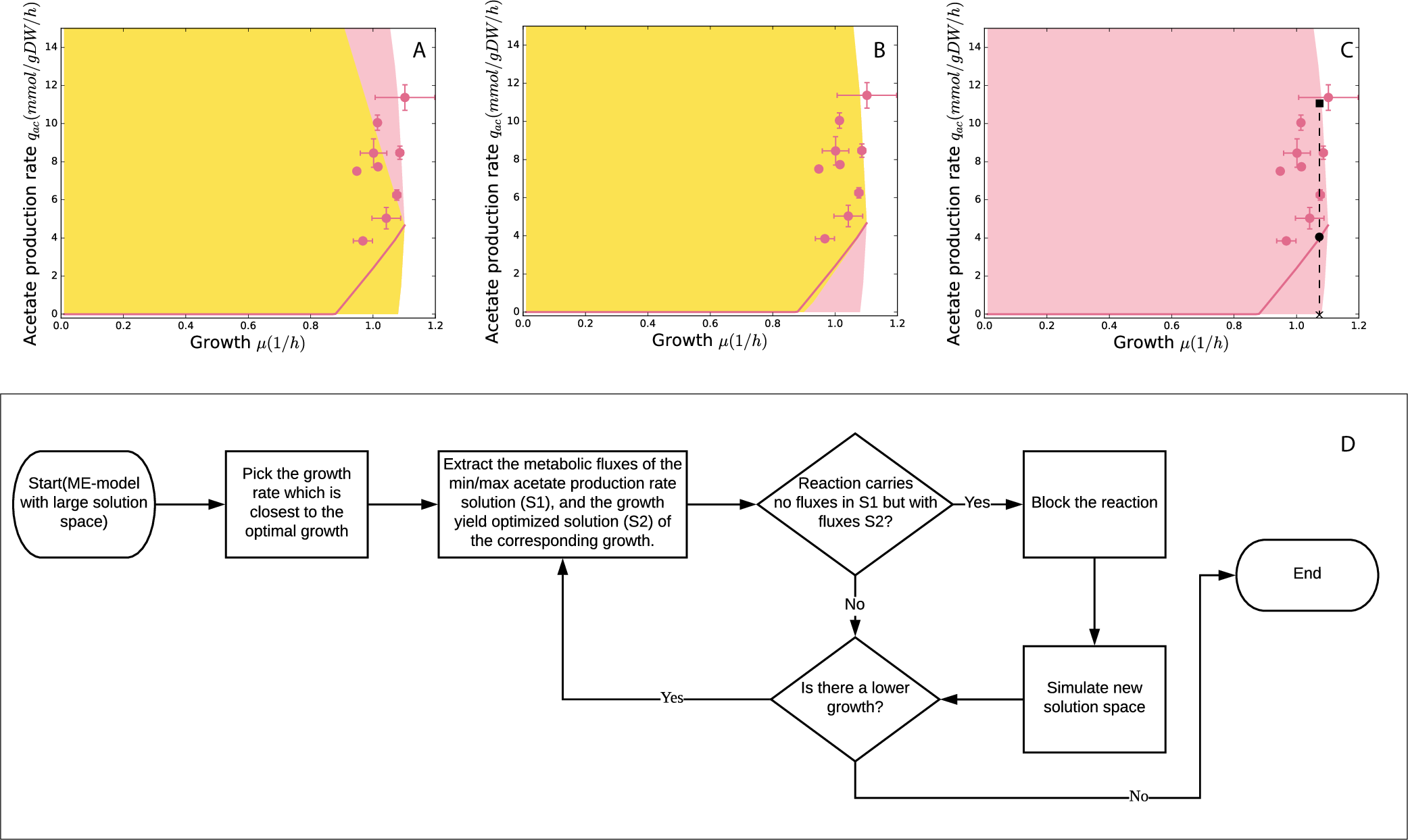

Supplement: S6 Fig — Narrowing in the feasible range of alternative suboptimal solutions by blocking some target reactions. The new solution space after the variation is shown as the yellow in (A) and (B), with the original solution space in pink. (A) 24 target reactions (S6 Table) that are blocked where maximum qacs in high μ get lower, where the upper edge of the yellow region is below the upper edge of the pink region. The activation of one of these 24 reactions thus corresponding to higher qac with lower Y. (B) 11 target reactions (S5 Table) corresponding to lower qac with lower Y, blocking those reactions will get the minimum qac (lower edge of the yellow region) closed to the Y-maximized qac solution. (C) The method of picking reactions to block: Looking for the reactions that are not activated in the yield-maximized solution but activated at the maximal and minimal of the μ–qac solution space, where the principal is to keep the Y-maximized solutions unchanged. (TIF) [file pcbi.1007066.s018.tif]

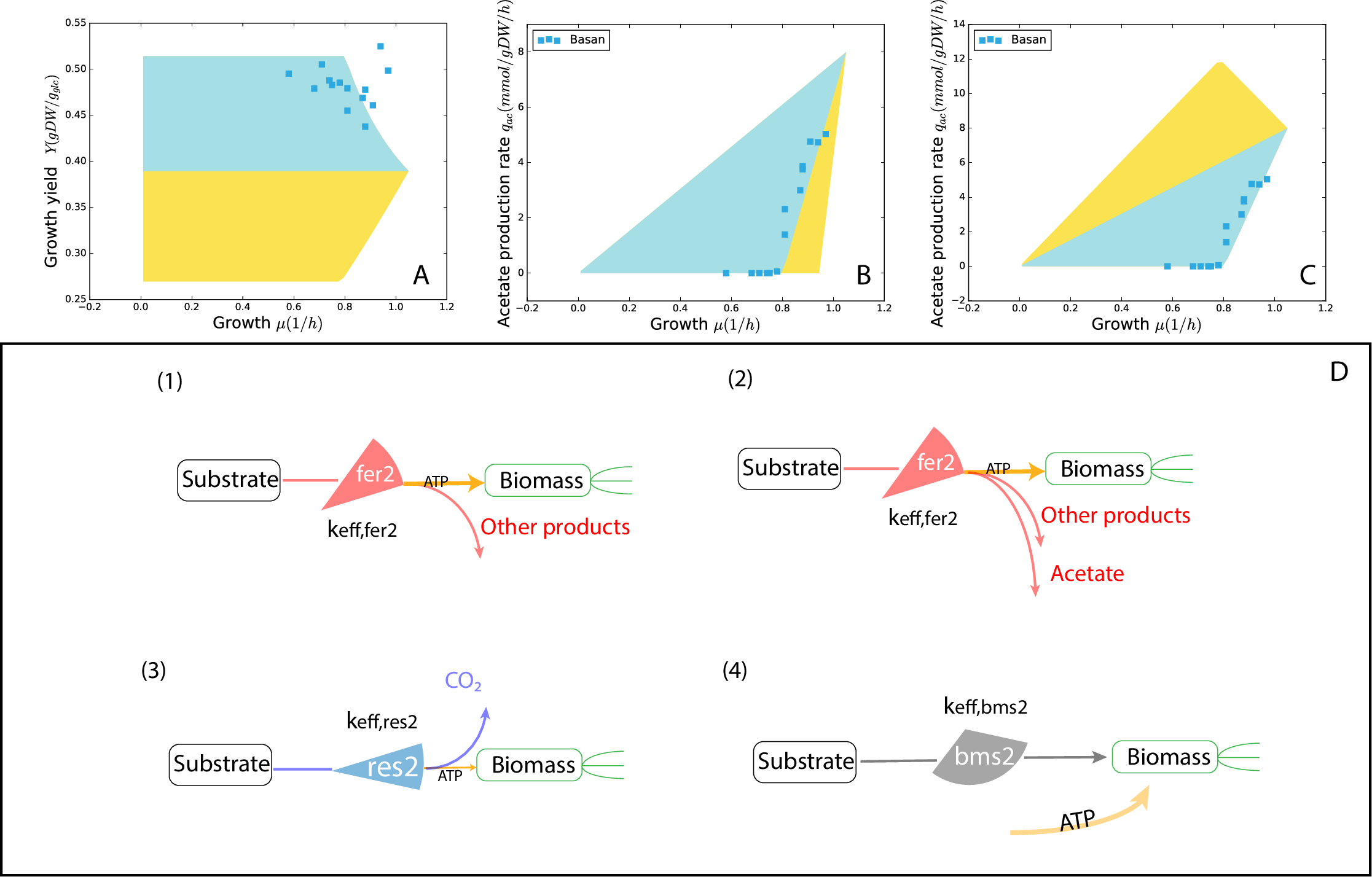

Supplement: S7 Fig — The expanded part of the solution space is shown as yellow in (A)–(C), compared to the original SSME-model solution spaces are in blue. (A) All added reactions ((1)-(4) in D) expand the solution space to include lower-Y solutions (B) Reactions (1) and (3) expand the solution space to low-qac at high μ. (C) Reactions (2) and (4) expand the solution space to high-qac across all μ. (D) Model reactions that are added in the SSME-model for expanding the original solution space, all those reactions are guaranteed not be activated in the Y-maximized solutions so that the Y-optimal solution remains the same to fit data from [5]. Reaction (1) corresponds to the reactions that would generate products other than acetate such as pyruvate excretion, lactate excretion, etc. Reaction (2) is representative to the reactions that would generate other products, but at the same time generating acetate, such as pyruvate formate lyase (PFL), which produce formate and acetyl-CoA (precursor of acetate) from pyruvate. Reaction (3) and (4) could both be referred from the futile cycle in energy production and consumption, where (3) are the reactions that are less efficient than the optimal pathway, such as the alternative reactions in ETC which are less efficient in transporting electrons, while (4) are the reactions that would waste more energy in the same growth comparing to the optimal state, such as the reactions that would cause proton leakage. (TIF) [file pcbi.1007066.s019.tif]
